# Multiplatform Morphometric Profiling of Whole-Brain, Cerebellar Subregional, and Thalamic Nuclei Alterations in Pediatric Migraine Without Aura

**DOI:** 10.3390/diagnostics16132085

**Published:** 2026-07-03

**Authors:** Adil Aytaç, Hilal Aydın, Emrah Akay

**Affiliations:** 1 Department of Radiology, Faculty of Medicine, Health Practice and Research Hospital, Balikesir University, 10145 Balıkesir, Turkey; 2 Department of Pediatric Neurology, Faculty of Medicine, Health Practice and Research Hospital, Balikesir University, 10145 Balıkesir, Turkey; 3 Department of Radiology, Bodrum State Hospital, 48400 Muğla, Turkey

**Keywords:** migraine disorders, magnetic resonance imaging, brain mapping, cerebellum, thalamus

## Abstract

**Objectives:** To investigate morphometric alterations associated with pediatric migraine without aura at the whole-brain, cerebellar subregional, and thalamic nuclei levels using a multiplatform magnetic resonance imaging (MRI) approach integrating the complementary strengths of different neuroimaging platforms. **Methods:** This retrospective study included 74 patients aged 12–17 years with migraine without aura and 70 control subjects with structurally normal cranial MRI findings. High-resolution isotropic three-dimensional T1-weighted MRI datasets were analyzed using a multiplatform segmentation workflow integrating vol2Brain, FreeSurfer, and 3D Slicer. Multiple comparisons were corrected using the Benjamini–Hochberg false discovery rate method. Significant volumetric measurements were subsequently normalized for total intracranial volume (TIV) and reanalyzed. **Results:** Compared with controls, patients with migraine exhibited lower bilateral amygdala and right thalamic volumes, as well as reduced cortical thickness in the right anterior insula, left anterior cingulate gyrus, and precuneus (all FDR-adjusted q < 0.05). Cerebellar analyses demonstrated reduced bilateral Lobule VI volume and gray matter volume, together with cortical thinning in the right Crus I and right Crus II (all FDR-adjusted q < 0.05). Thalamic nuclei analyses revealed lower volumes of the bilateral mediodorsal and pulvinar nuclei and the right ventral anterior and ventral posterolateral nuclei (all FDR-adjusted q < 0.05). All volumetric findings remained significant after TIV normalization. **Conclusions:** By integrating whole-brain morphometry with detailed cerebellar subregional and thalamic nuclei analyses, this study expands conventional morphometric assessment and provides a more integrated perspective on structural brain alterations associated with pediatric migraine without aura.

## 1. Introduction

Pediatric migraine is a common neurological disorder characterized by recurrent headache attacks during childhood [1]. Its pathophysiology is believed to involve genetic susceptibility, activation of the trigeminovascular system, alterations in cortical excitability, neuroinflammatory mechanisms, and neurophysiological processes within the central nervous system [1]. With a reported prevalence of approximately 8–15% in the pediatric population and an increasing frequency with advancing age during childhood and adolescence, migraine is recognized as a significant cause of neurological morbidity that can substantially affect academic performance, daily activities, and psychosocial functioning [2]. The pediatric period also represents a critical stage of neurodevelopment, during which synaptic pruning, white matter maturation, cortical reorganization, and the integration of large-scale neural networks continue to occur [3]. Consequently, migraine occurring during this period of active neurodevelopment may be associated with global or regional morphometric characteristics of the developing brain [4].

Recent neuroimaging and neurophysiological studies suggest that pediatric migraine cannot be explained solely by vascular mechanisms and should instead be considered a complex neurobiological disorder involving pain modulation, sensory processing, affective regulation, and cortical–subcortical integration [5,6,7]. Several functional and structural neuroimaging studies have reported structural and functional alterations in brain regions associated with pain processing, sensory integration, emotional regulation, and cognitive functions in patients with migraine [8,9]. Among these regions, the anterior cingulate cortex, insula, thalamus, amygdala, basal ganglia, brainstem, cerebellum, and thalamic nuclei have emerged as anatomically relevant structures in migraine pathophysiology [5,7,8,9].

The functional relevance of these neuroanatomical regions can be further understood in the context of peripheral and central sensitization. Recurrent activation of trigeminovascular nociceptive pathways may increase the responsiveness of peripheral trigeminal afferents and subsequently facilitate central amplification within second- and higher-order nociceptive neurons. This process may contribute to enhanced pain sensitivity, abnormal sensory gain, and altered pain modulation beyond the ictal phase [7,10,11]. Recent quantitative sensory evidence showing reduced pressure pain thresholds not only in trigeminal but also in extra-trigeminal areas in episodic and chronic migraine further supports the concept that sensitization in migraine may involve widespread pain-processing abnormalities rather than being restricted to cranial nociceptive pathways [10]. Within this framework, the anterior insula and anterior cingulate cortex are particularly relevant because they represent key nodes of the salience network, which plays a central role in interoceptive awareness, affective pain processing, and cognitive–emotional integration, whereas the amygdala contributes to emotional regulation and descending pain modulation [5,7,8,9]. Repeated nociceptive input and altered sensory processing may therefore be associated with activity-dependent functional reorganization across distributed cortical, limbic, and subcortical networks, providing a biological rationale for investigating morphometric alterations in these regions.

Beyond sensitization, impaired habituation to repetitive sensory stimulation represents one of the most consistently reported neurophysiological abnormalities in migraine [11]. Rather than showing the expected reduction in response amplitude during repeated stimulation, individuals with migraine often demonstrate deficient habituation across sensory modalities, suggesting altered cortical excitability, impaired sensory filtering, and dysfunctional thalamocortical communication [11]. These mechanisms are relevant to structural neuroimaging because the thalamus is not a passive relay structure but a set of anatomically and functionally distinct nuclei involved in nociceptive transmission, sensory integration, and cortical network regulation [7,9,11]. The cerebellum may also contribute to migraine pathophysiology through its non-motor roles in sensory prediction, pain modulation, autonomic regulation, and cerebellar–thalamocortical interactions [7,10,11,12]. Accordingly, contemporary models of migraine support the evaluation of structural alterations across multiple interconnected neuroanatomical levels, including whole-brain morphometry, cerebellar subregions, and thalamic nuclei, rather than focusing exclusively on isolated regional findings.

The interpretation of morphometric findings in pediatric migraine is complicated by age-related neurodevelopmental variability. Ongoing cortical remodeling, white matter maturation, and neuroplasticity processes may influence regional brain volumes and cortical thickness measurements independently of disease-related effects [3,4,6]. Consequently, morphometric neuroimaging studies in pediatric migraine have yielded heterogeneous findings, and no clear consensus has yet been reached regarding the extent and distribution of neuroanatomical alterations associated with the disorder [4,5,9,13,14]. This variability may be attributable to differences in migraine subtypes, age distribution, sex-related factors, imaging methodologies, and developmentally heterogeneous study populations [3,4,6,13,14].

Volumetric analyses, voxel-based morphometry, cortical thickness measurements, and quantitative segmentation techniques are widely used to investigate brain alterations associated with migraine [4,5,9,13,14]. Previous studies have reported morphometric findings involving gray and white matter structures, cortical volume and thickness, subcortical regions, and the cerebellum. However, the anatomical distribution and characteristics of these findings have varied considerably across studies [4,5,9,12,13,14]. Furthermore, while many investigations have focused on specific brain regions, studies evaluating the brain as a whole remain relatively limited.

The cerebellum is increasingly recognized as a structure involved not only in motor coordination but also in pain modulation, sensory integration, autonomic regulation, and cognitive-affective processing [7,12,15,16]. In particular, the posterior cerebellar lobules have attracted attention in migraine research because of their involvement in sensory and affective functions [12,15,17]. Nevertheless, studies examining individual cerebellar subregions in pediatric migraine are relatively scarce, and the cerebellum has often been evaluated as a single unified structure.

The thalamus is a major subcortical structure involved in sensory information relay, pain modulation, and a variety of sensorimotor functions [7,16,18]. However, it consists of numerous nuclei with distinct anatomical and functional characteristics. Accordingly, nucleus-level volumetric analyses may provide a more detailed assessment of regional volumetric alterations that could remain undetected when evaluating total thalamic volume alone. Despite this potential advantage, studies specifically investigating thalamic nuclei in pediatric migraine are limited, and these structures have rarely been comprehensively assessed alongside other brain regions [5,9,12,18,19].

Advanced automated neuroimaging processing pipelines and quantitative segmentation platforms are widely employed in contemporary computational neuroanatomy research. These approaches enable volumetric measurements to be obtained in a more standardized, reproducible, and less observer-dependent manner [5,14,19,20,21]. However, differences in atlases and processing workflows across segmentation platforms may contribute to variability in volumetric measurements, particularly in pediatric populations [3,5,14,20,21]. Therefore, hybrid workflows that integrate the complementary strengths of multiple platforms and incorporate expert validation may facilitate more reliable morphometric assessments [20,21,22].

To the best of our knowledge, studies simultaneously evaluating whole-brain morphometry, cerebellar subregional morphometry, and thalamic nuclei volumetry in pediatric migraine remain scarce. Moreover, most existing investigations have been based on adult cohorts, isolated regional analyses, or single-platform segmentation approaches [4,13,21]. To address this gap, the present study evaluated whole-brain morphometric measurements, cerebellar subregions, and thalamic nuclei volumes in pediatric patients aged 12–17 years with migraine without aura using an integrated multiplatform segmentation workflow that combined the complementary strengths of vol2Brain (version 1.0, release 23 November 2021; Universitat Politècnica de València, Valencia, Spain), FreeSurfer (version 7.4.1; Martinos Center for Biomedical Imaging, Massachusetts General Hospital, Boston, MA, USA), and 3D Slicer (version 5.6.1; Brigham and Women’s Hospital, Harvard Medical School, Boston, MA, USA). We hypothesized that pediatric migraine may be associated with region-specific morphometric alterations distributed across cortical, cerebellar, and thalamic structures rather than with changes confined to a single anatomical region. This comprehensive approach may contribute to a more holistic characterization of migraine-related morphometric alterations in the pediatric population and further expand current knowledge in this field.

## 2. Methods

### 2.1. Study Design and Ethical Approval

This single-center, retrospective, observational neuroimaging study was conducted at Balıkesir University Faculty of Medicine. The study protocol was approved by the Balıkesir University Non-Interventional Health Sciences Research Ethics Committee (Approval No: 2024/179; Date: 5 November 2024). All study procedures were performed in accordance with the principles of the Declaration of Helsinki, and the study was reported following the Strengthening the Reporting of Observational Studies in Epidemiology guidelines for observational research. Written informed consent was obtained from all participants or their legal guardians before inclusion in the study. Institutional Picture Archiving and Communication System (PACS) records were retrospectively reviewed to identify pediatric patients diagnosed with migraine without aura who underwent cranial magnetic resonance imaging (MRI) between January 2017 and October 2024.

### 2.2. Study Population

In this retrospective study, 74 patients aged 12–17 years with migraine without aura and 70 control subjects were included. Inclusion and exclusion criteria were predefined to reduce neurodevelopmental heterogeneity, limit age-related neuroanatomical variation, and minimize potential confounding factors that could influence volumetric measurements. The restriction of the study population to individuals aged 12–17 years was intended to reduce the effects of rapid global brain growth, pronounced cortical reorganization, and age-dependent neurodevelopmental variability characteristic of early childhood, thereby enabling a more reliable assessment of morphometric alterations associated with migraine without aura [23,24,25,26]. Furthermore, this age range represents a critical neurodevelopmental period during which cortical maturation, white matter reorganization, cerebellar development, and the refinement of cortical–subcortical networks remain ongoing, while neurodevelopmental variability is relatively less pronounced than in younger pediatric populations [23,24,25,26]. All MRI examinations were performed at a single institution using standardized imaging protocols.

The inclusion criteria for the migraine without aura group were as follows:

• Age between 12 and 17 years;
• A diagnosis of migraine without aura according to the criteria defined in the third edition of the International Classification of Headache Disorders (ICHD-3) [27];
• Confirmation of the migraine diagnosis by a pediatric neurologist with 10 years of experience in pediatric headache disorders based on clinical history, headache characteristics, attack pattern, and associated symptoms;
• Normal neurological examination findings;
• Availability of a diagnostically adequate cranial MRI examination acquired at the same institution;
• Availability of high-resolution isotropic three-dimensional (3D) T1-weighted images suitable for volumetric analysis;
• Absence of structural, developmental, or signal abnormalities on conventional MRI that could affect volumetric assessment;
• Complete clinical and imaging data.

The inclusion criteria for the control group were as follows:

• Age between 12 and 17 years;
• Undergoing cranial MRI because of syncope, transient alteration of consciousness, or benign nonfocal neurological complaints unrelated to migraine or other primary headache disorders;
• No history of migraine or any other primary headache disorder;
• Normal neurological examination findings;
• Structurally normal cranial MRI findings;
• No history of epilepsy, neurodevelopmental disorders, psychiatric disease, or chronic systemic illness;
• No history of any neurological disorder requiring regular neurological follow-up;
• Availability of a diagnostically adequate cranial MRI examination acquired at the same institution.

All cranial MRI examinations in the control group were independently re-evaluated by a neuroradiologist with 9 years of experience. This secondary review was performed to minimize the risk of overlooked imaging abnormalities and to enhance the homogeneity of the control cohort. This approach represents a commonly adopted methodological strategy in pediatric neuroimaging studies aimed at establishing a clinically screened reference cohort with structurally normal brain MRI findings [28,29,30]. Accordingly, every effort was made to minimize potential confounding factors that could influence brain morphology within the control group.

The exclusion criteria for the migraine group were as follows:

• Diagnosis of migraine with aura or documentation of aura symptoms in the clinical records;
• Diagnosis of chronic migraine according to ICHD-3 criteria;
• Reporting ≥ 15 headache days per month during the preceding three months;
• Diagnosis of, or clinical suspicion for, a secondary headache syndrome;
• Diagnosis of, or clinical suspicion for, medication-overuse headache;
• History of long-term prophylactic migraine treatment;
• Regular use of antiepileptic drugs, antidepressants, antipsychotics, psychostimulants, long-term sedatives, or other medications that could affect central nervous system function;
• Use of hormonal therapy;
• Presence of endocrine disorders, hormonal diseases, or systemic conditions that could influence pubertal development;
• Cases in which the migraine subtype could not be reliably verified from clinical records.

The exclusion criteria applicable to both groups were as follows:

• Presence of a chronic systemic disease requiring long-term medical treatment;
• Known vascular or demyelinating disease;
• History of previous neurosurgical intervention;
• History of traumatic brain injury;
• Presence of congenital brain malformations or incidental intracranial structural lesions;
• Incomplete clinical or imaging data;
• MRI examinations performed outside the institution;
• Imaging datasets unsuitable for reliable automated segmentation because of substantial motion artifacts, inadequate image quality, or failure to achieve anatomical verification during the quality control process.

**Figure 1:**
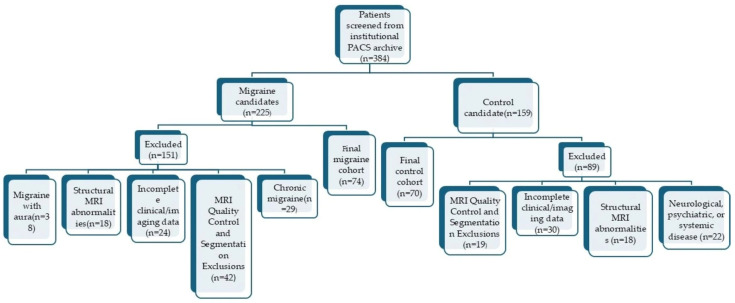
Flowchart showing patient selection, eligibility assessment, exclusion criteria, and final cohort formation. Of 384 subjects screened from the institutional PACS archive, 74 patients with migraine without aura and 70 controls with structurally normal brain MRI findings met all study criteria and were included in the final volumetric analyses. Detailed reasons for exclusion are provided within the flowchart.

The final study cohort obtained after applying the inclusion and exclusion criteria is illustrated in the participant flowchart presented in Figure 1.

### 2.3. Magnetic Resonance Imaging Protocol

All MRI examinations were performed using a 1.5-T MRI system (Ingenia, Philips Medical Systems, Best, The Netherlands) equipped with a standard 16-channel head coil. A retrospective review of the technical records revealed no major hardware or software upgrades during the study period that could have influenced volumetric measurements. Therefore, the imaging platform was considered stable throughout the study period, and all 3D T1-weighted images were assumed to have been acquired under comparable technical conditions.

The MRI acquisition parameters used in the cranial imaging protocol were as follows:

• Axial T1-weighted spin-echo (SE): repetition time/echo time (TR/TE), 450/15 ms; field of view (FOV), 230 mm; slice thickness, 5 mm; matrix, 308 × 183.
• Axial fat-saturated T1-weighted SE: TR/TE, 633/15 ms; FOV, 230 mm; slice thickness, 5 mm; matrix, 308 × 183.
• Axial T2-weighted turbo spin-echo (TSE): TR/TE, 5240/100 ms; FOV, 230 mm; slice thickness, 5 mm; matrix, 384 × 237.
• Coronal fluid-attenuated inversion recovery (FLAIR): TR/TE, 11,000/130 ms; FOV, 230 mm; slice thickness, 5 mm; matrix, 256 × 157.
• Coronal T2-weighted TSE: TR/TE, 3027/100 ms; FOV, 200 mm; slice thickness, 3 mm; matrix, 336 × 217.
• Coronal T1-weighted inversion recovery: TR/TE, 3079/15 ms; FOV, 200 mm; slice thickness, 3.5 mm; matrix, 336 × 211.
• Coronal FLAIR: TR/TE, 11,000/130 ms; FOV, 230 mm; slice thickness, 3 mm; matrix, 256 × 157.
• 3D FLAIR: TR/TE, 4800/315 ms; FOV, 250 mm; slice thickness, 1.04 mm; matrix, 216 × 218.
• Axial diffusion-weighted imaging (DWI): b-values, 0 and 1000 s/mm^2^; FOV, 230 mm; slice thickness, 5 mm; matrix, 152 × 106.
• Isotropic 3D T1-weighted imaging: TR/TE, 2500/46 ms; FOV, 230 mm; slice thickness, 1.0 mm; matrix, 256 × 256.

No contrast agent was administered in any case. All segmentation procedures and volumetric analyses were performed using the high-resolution isotropic 3D T1-weighted images. The remaining MRI sequences were used exclusively for structural assessment, quality control, and anatomical verification.

### 2.4. Magnetic Resonance Imaging Quality Control Pipeline

All imaging datasets underwent a multistep quality control procedure before volumetric analysis and automated segmentation. In the first stage, DICOM datasets were retrospectively reviewed for technical integrity. Examinations containing incomplete image series, corrupted image data, or incompatible acquisition parameters were excluded. In the second stage, all images were visually assessed for motion artifacts, signal degradation, image blurring, and reconstruction errors. Studies in which anatomical structures could not be reliably evaluated were excluded from further analysis.

Before volumetric processing, all imaging datasets were converted from DICOM to Neuroimaging Informatics Technology Initiative (NIfTI) format. Following conversion, image orientation, voxel dimensions, spatial resolution, matrix configuration, and header integrity were verified. Adequate intracranial coverage was also confirmed for all examinations. Only datasets that successfully passed all quality-control steps were forwarded to the automated segmentation and subsequent expert validation procedures.

Whole-brain volumetric measurements and cerebellar subregional analyses were performed using vol2Brain. Thalamic nuclei segmentation was performed using FreeSurfer. All segmentation outputs were subsequently visualized and subjected to quality control using 3D Slicer. Anatomical accuracy was assessed by the same experienced neuroradiologist in three orthogonal planes (axial, coronal, and sagittal), and the correspondence between segmentation boundaries and the individual 3D T1-weighted images was verified.

To evaluate methodological consistency, a secondary quality control analysis was performed in a randomly selected subgroup comprising 15% of the study population. Within this subgroup, the automated segmentation results were independently reviewed in 3D Slicer by a neuroradiologist with 8 years of experience. All quality control assessments were conducted with reviewers blinded to clinical group allocation. Examinations containing substantial segmentation errors or other issues that could compromise the reliability of volumetric measurements were excluded from the final analysis.

**Figure 2:**
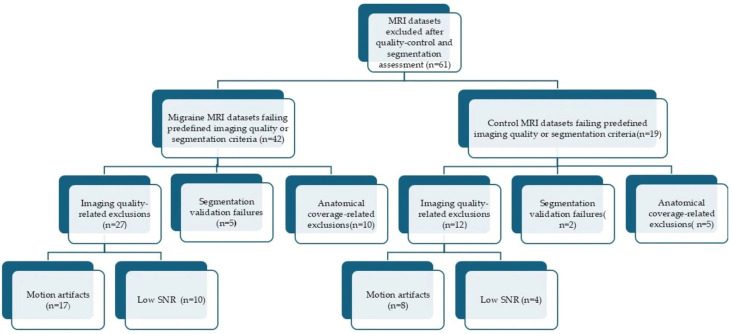
Flowchart illustrating the MRI quality-control and segmentation validation workflow. A total of 61 MRI datasets (42 migraine candidates and 19 control candidates) were excluded based on predefined image quality and segmentation assessment criteria. Exclusion reasons included motion artifacts, low signal-to-noise ratio, incomplete anatomical coverage, limited field of view, and segmentation validation failures. Only datasets meeting all quality-control requirements were included in the final volumetric analyses.

The MRI quality control workflow, segmentation verification procedures, and multiplatform neuroimaging processing pipeline are summarized in Figure 2.

### 2.5. Neuroimaging Processing Workflow

A multiplatform neuroimaging workflow was implemented to enable the integrated assessment of whole-brain volumetry, cerebellar subregional morphometry, and thalamic nuclei segmentation [20,21,22,31]. Platform selection was based on the specific methodological strengths of each software package and its suitability for the corresponding neuroanatomical analysis. vol2Brain was used for whole-brain morphometric and cerebellar assessments because of its automated atlas-based segmentation framework and comprehensive volumetric parcellation capabilities. FreeSurfer was selected for thalamic nuclei analyses because its dedicated Bayesian thalamic nuclei segmentation module enables detailed nucleus-level volumetric assessment. 3D Slicer was employed as an independent visualization and quality-control environment for expert anatomical verification of all automated segmentation outputs before inclusion in the final analyses. The vol2Brain platform was selected for whole-brain and cerebellar analyses because it provides detailed automated volumetric outputs for a large number of cortical, subcortical, and cerebellar anatomical structures and facilitates comprehensive morphometric evaluation of cerebellar subregions [32]. Owing to the small anatomical size and complex structural organization of the thalamic nuclei, thalamic segmentation was performed using a FreeSurfer-based approach that incorporates a histology-informed Bayesian atlas framework [33].

The segmentation masks generated by FreeSurfer were subsequently imported into 3D Slicer for anatomical verification. 3D Slicer was selected for quality control purposes because it enables segmentation masks to be overlaid onto the native high-resolution 3D T1-weighted images and allows simultaneous evaluation in the axial, coronal, and sagittal planes [22,31]. Consequently, segmentation, quality control, and volumetric quantification were performed within an integrated, traceable, and anatomically verifiable analysis framework [20,21,22,31]. All automated segmentation outputs subsequently underwent expert anatomical verification and quality-control assessment before inclusion in the final statistical analyses.

#### 2.5.1. vol2Brain Processing Pipeline

Whole-brain volumetric measurements and cerebellar subregional analyses were performed using vol2Brain [32]. Vol2Brain is an image-processing platform that integrates high-resolution voxel-based morphometry with automated volumetric quantification algorithms based on SPM12 and CAT12 frameworks [34]. This system enables fully automated and reproducible processing of high-resolution T1-weighted MRI datasets.

All MRI datasets were retrieved from the institutional archive in DICOM format. To optimize segmentation accuracy, only high-resolution 3D T1-weighted images with isotropic voxel dimensions of ≤1 mm^3^ were included in the analysis [35]. After completion of the quality control procedure and anonymization, imaging datasets were converted to NIfTI format using the dcm2niix conversion tool included in the MRIcroGL version 1.2.20220720 (Chris Rorden, University of South Carolina, Columbia, SC, USA) [36].

Cortical and subcortical parcellation was performed using the non-local multi-atlas patch-based segmentation approach integrated into the vol2Brain processing pipeline [32,33]. This multi-atlas framework accounts for interindividual neuroanatomical variability and has been widely applied in the automated analysis of brain images acquired in developmental populations [37]. Following segmentation, automated volumetric measurements were generated for each anatomical structure.

Whole-brain volumetric analysis included assessment of total gray matter volume, total white matter volume, total brain volume, brainstem volume, cerebrospinal fluid (CSF) compartments, and total intracranial volume. In addition, the vol2Brain platform provided automated morphometric measurements for a total of 135 anatomical regions encompassing cortical, subcortical, cerebellar, and CSF-related structures [32,38].

For cerebellar morphometric analysis, 12 cerebellar subregions were evaluated, including the cerebellar hemispheres, vermian regions, and posterior cerebellar lobules. Volumetric measurements for each subregion were analyzed independently.

All automated segmentation outputs underwent anatomical verification using 3D Slicer. Segmentation boundaries and anatomical alignment were assessed on the individual 3D T1-weighted images by the same experienced neuroradiologist. Segmentations deemed inadequate for anatomical verification were excluded from the analysis.

Representative examples of the whole-brain volumetric output and cerebellar subregional analysis obtained from a patient in the pediatric migraine without aura group are presented in Supplementary Material S1 and Supplementary Material S2, respectively.

**Figure 3:**
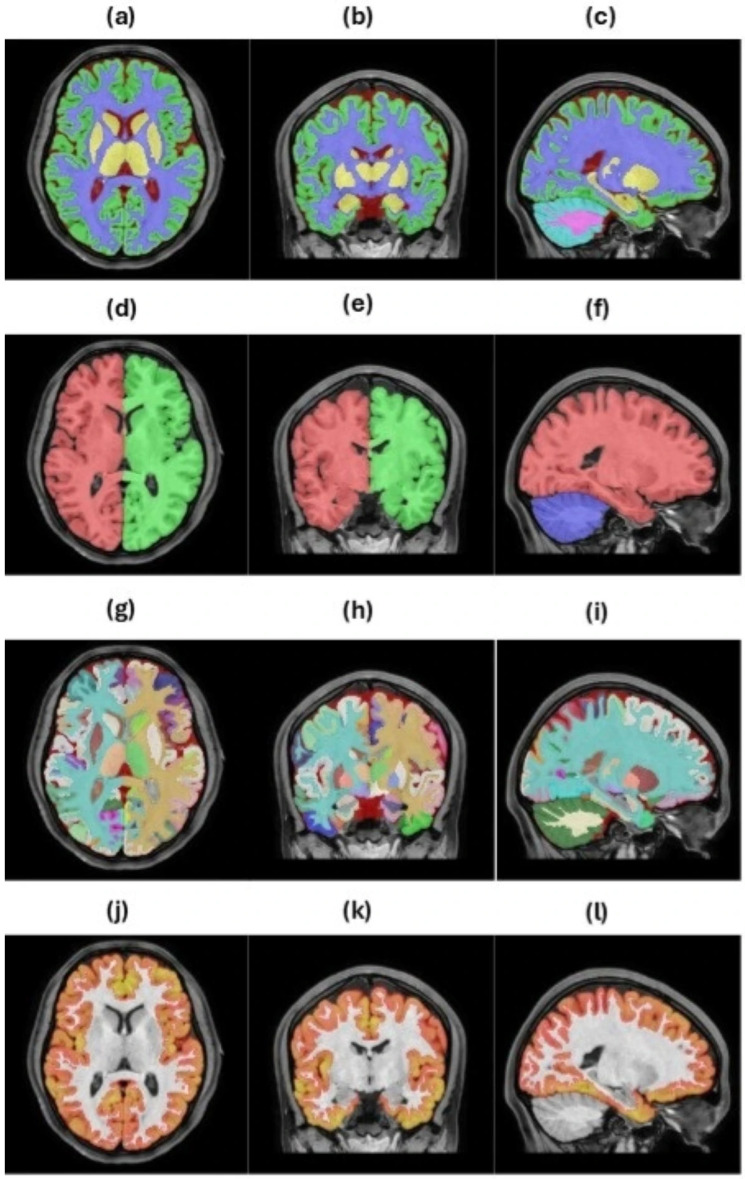
Representative automated brain segmentation and morphometric analysis generated using the Vol2Brain platform from a high-resolution 3D T1-weighted MRI examination. Panels (**a**–**c**) demonstrate tissue segmentation in the axial, coronal, and sagittal planes, including gray matter, white matter, cerebrospinal fluid (CSF), and deep gray matter structures. Panels (**d**–**f**) show macrostructural segmentation of major anatomical brain regions. Panels (**g**–**i**) illustrate detailed cortical and subcortical parcellation, while panels (**j**–**l**) depict cortical thickness mapping using color-coded surface visualization.

**Figure 4:**
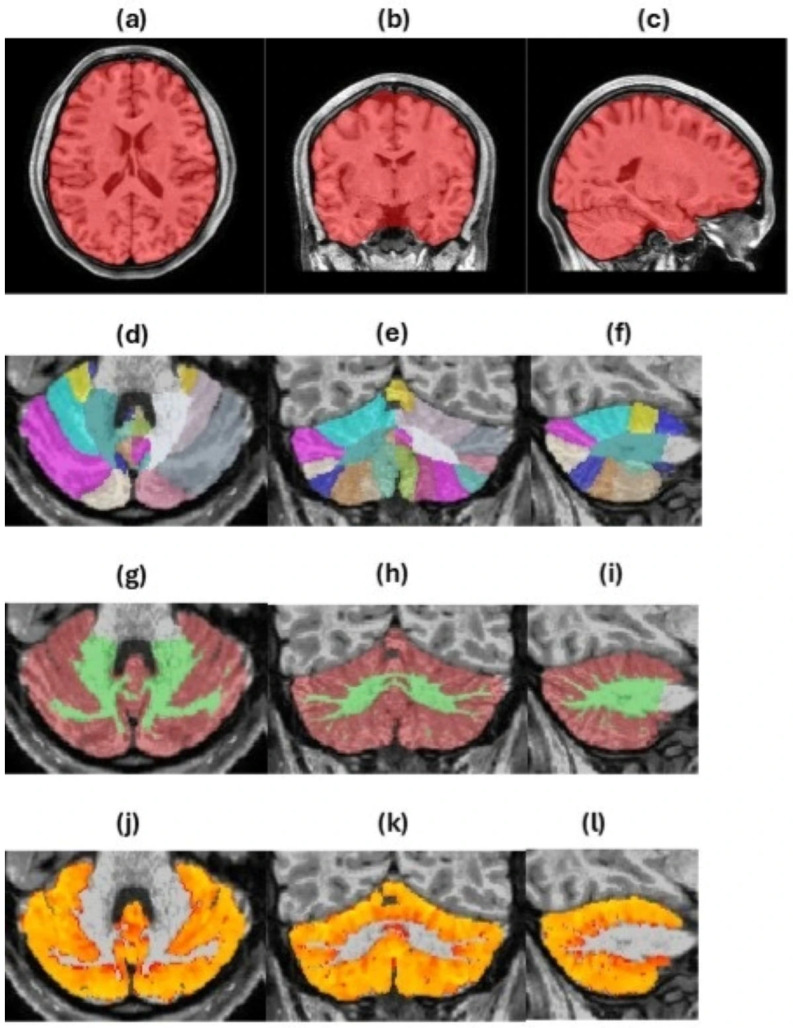
Representative cerebellar morphometric analyses generated using the Vol2Brain platform from a high-resolution 3D T1-weighted MRI examination. Panels (**a**–**c**) demonstrate intracranial cavity segmentation in the axial, coronal, and sagittal planes. Panels (**d**–**f**) illustrate automated cerebellar lobule segmentation with color-coded anatomical parcellation. Panels (**g**–**i**) present cerebellar tissue segmentation, including gray matter and white matter compartmentalization. Panels (**j**–**l**) depict cerebellar cortical thickness mapping using color-coded surface visualization.

Representative examples of automated cortical and subcortical parcellation performed using the vol2Brain processing pipeline on high-resolution isotropic 3D T1-weighted MRI datasets are presented in Figure 3. Automated segmentation of cerebellar subregions derived from the same processing workflow is illustrated in Figure 4.

#### 2.5.2. FreeSurfer Processing Pipeline

Thalamic nuclei were analyzed separately because of their small anatomical volumes and susceptibility to partial volume effects. All segmentations underwent an advanced visual quality control procedure.

Thalamic nuclei segmentation was performed using FreeSurfer software (version 7.4.1; Martinos Center for Biomedical Imaging, Massachusetts General Hospital, Boston, MA, USA) running within a Docker-based Ubuntu environment [39]. All analyses were conducted automatically using the FreeSurfer recon-all processing pipeline applied to high-resolution isotropic 3D T1-weighted MRI datasets. This pipeline includes bias-field correction, intensity normalization, skull stripping, linear registration to Talairach space, subcortical segmentation, and cortical surface reconstruction [40].

Thalamic nuclei segmentation was generated using the Bayesian atlas-based thalamic nuclei segmentation module integrated into the FreeSurfer platform. This approach enables automated parcellation of the thalamus into anatomically distinct subnuclear structures using high-resolution probabilistic atlases derived from histological data [33]. The classification of the thalamic nuclei evaluated in the present study is presented in Table 1. A detailed description of the computational workflow used for FreeSurfer-based thalamic nuclei segmentation is provided in Supplementary Material S3.

**Figure 5:**
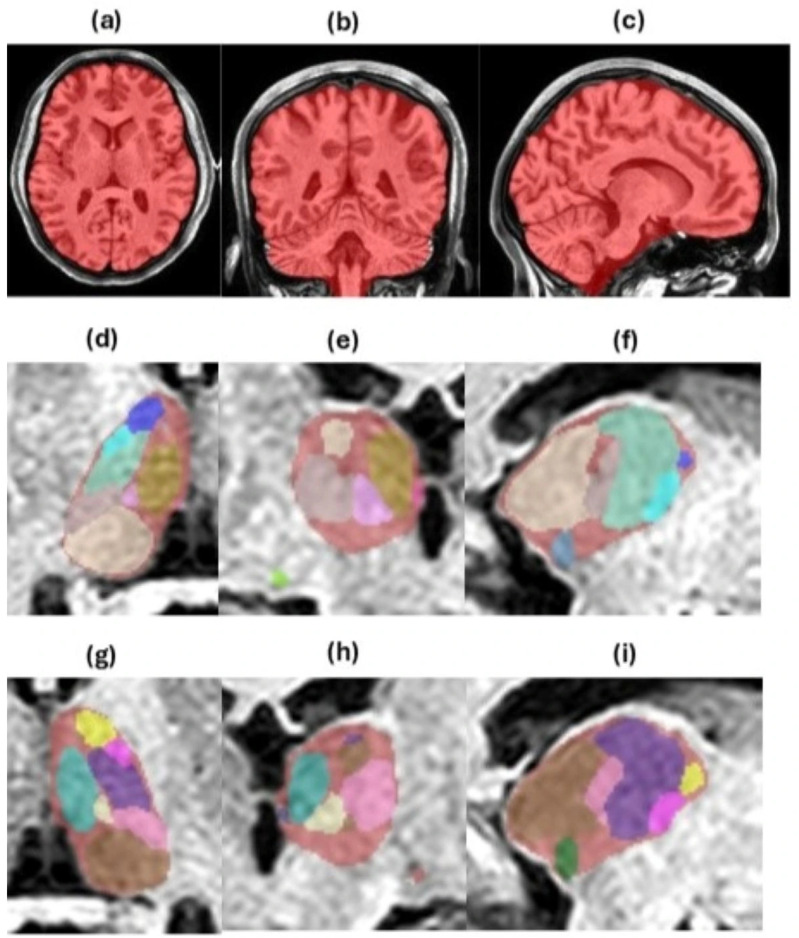
Representative automated thalamic nuclei segmentation obtained from high-resolutionthree-dimensional (3D) T1-weighted magnetic resonance images using the FreeSurfer thalamic nuclei module. Panels (**a**–**c**) demonstrate intracranial cavity segmentation in the axial, coronal, and sagittal planes. Panels (**d**–**f**) illustrate color-coded segmentation of the left thalamic nuclei across different anatomical planes, whereas panels (**g**–**i**) show the corresponding segmentation of the right thalamic nuclei. Distinct colors represent anatomically defined thalamic nuclei.

**Figure 6:**
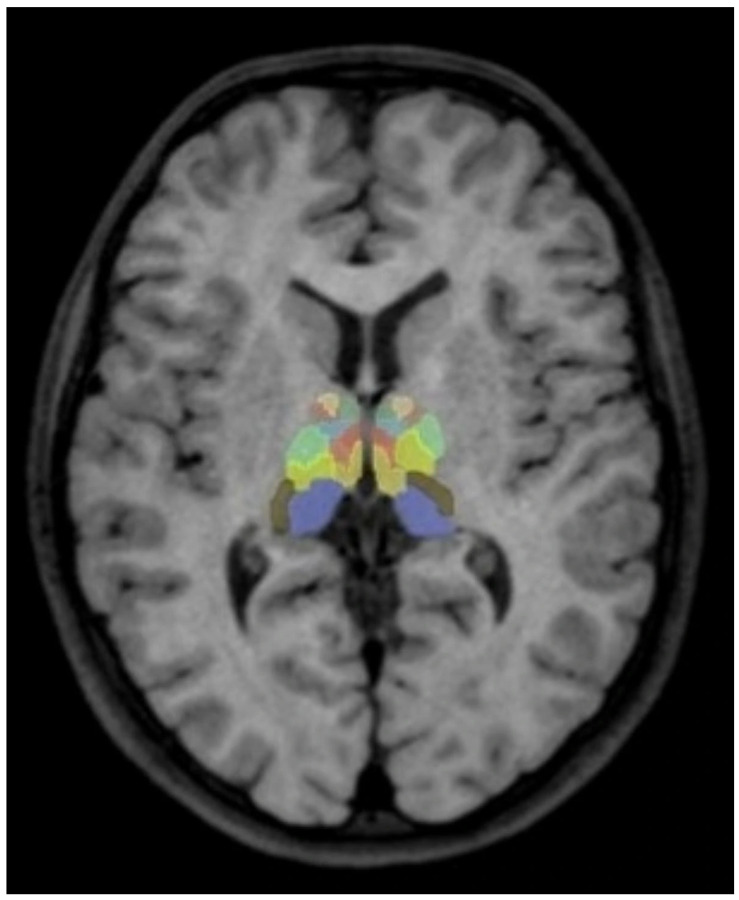
Representative axial three-dimensional (3D) T1-weighted magnetic resonance image demonstrating automated color-coded segmentation of bilateral thalamic nuclei using the FreeSurfer thalamic nuclei module. Distinct colors correspond to anatomically defined thalamic nuclei included in the volumetric analysis: anteroventral nucleus (AVN, orange), mediodorsal nucleus (MN, salmon-red), ventrolateral anterior nucleus (VLAN, yellow), ventral anterior nucleus (VAN, light green), ventral posterolateral nucleus (VPLN, dark green), lateral geniculate nucleus (LGN, cyan), medial geniculate nucleus (MGN, light blue), intralaminar nuclei (ISN, purple), and pulvinar nucleus (PN, dark blue).

Thalamic nuclei segmentation was performed separately for the right and left hemispheres. Given the small anatomical size of the thalamic nuclei and their susceptibility to partial volume effects, all segmentation outputs were re-evaluated in 3D Slicer and subjected to anatomical verification [41]. Representative examples of automated thalamic nuclei segmentation are presented in Figure 5, whereas the anatomical distribution of the thalamic nuclei evaluated in this study is illustrated in Figure 6.

#### 2.5.3. Visual Validation and Volumetric Analysis Using 3D Slicer

Segmentation outputs generated by the FreeSurfer processing pipeline were converted from MGZ format to NIfTI format and subsequently imported into 3D Slicer for further evaluation [33,41]. All segmentation masks were overlaid onto the native high-resolution 3D T1-weighted images and reassessed in the axial, coronal, and sagittal planes. During the quality control process, anatomical correspondence, boundary integrity, potential segmentation errors, and bilateral symmetry were systematically evaluated.

The anatomical accuracy of each segmentation was assessed by an experienced neuroradiologist through inspection across coordinates corresponding to the X-, Y-, and Z-axes in the axial, coronal, and sagittal planes. Spatial correspondence between the segmentation masks and the individual 3D T1-weighted images was visually verified. All evaluations were performed with the reviewer blinded to clinical group allocation.

Volumetric measurements were obtained using the Segment Statistics module integrated within the 3D Slicer platform. Volumes for each thalamic nucleus were automatically calculated in cubic millimeters (mm^3^), and measurements from the right and left hemispheres were analyzed separately. Examinations with inadequate anatomical verification or substantial segmentation errors were excluded from the final analysis. This validation strategy was implemented to ensure anatomical accuracy, minimize segmentation-related bias, and improve the reliability of segmentation-derived morphometric measurements.

**Figure 7:**
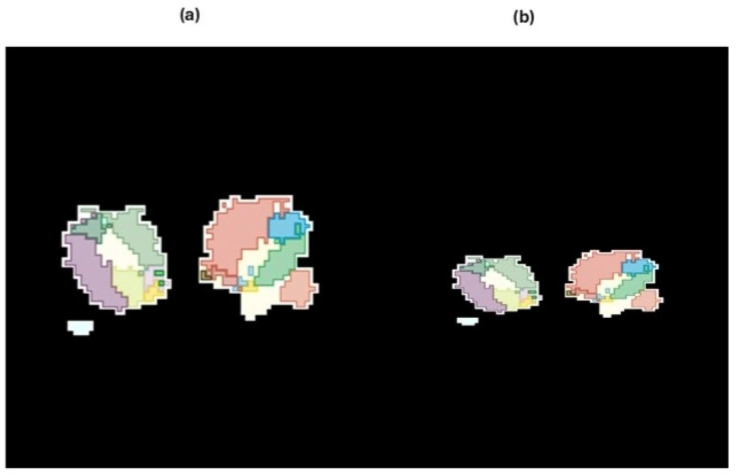

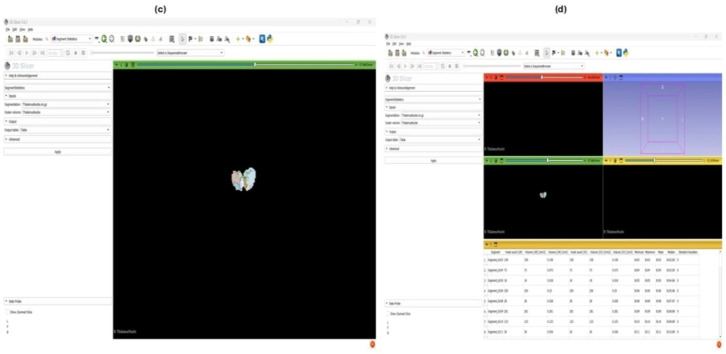
Representative 3D Slicer-based anatomical verification and quantitative volumetric analysis of automated thalamic nuclei segmentation. Panels (**a**,**b**) demonstrate color-coded bilateral thalamic nuclei segmentation masks at different slice levels following FreeSurfer-based automated segmentation. Panel (**c**) shows visualization of the imported segmentation masks within the 3D Slicer environment for anatomical verification and quality control. Panel (**d**) illustrates quantitative volumetric assessment using the Segment Statistics module, through which individual thalamic nuclear volumes were automatically calculated and exported for statistical analysis.

Representative images demonstrating the 3D Slicer–based anatomical validation procedure and quantitative volumetric analysis of the automated segmentations are presented in Figure 7.

### 2.6. Statistical Analysis

All statistical analyses were performed using IBM SPSS Statistics for Windows (version 29.0; IBM Corp., Armonk, NY, USA) and RStudio (version 4.3.3; Posit Software, PBC, Boston, MA, USA). The distribution of continuous variables was assessed using the Shapiro–Wilk test. Normally distributed variables are presented as mean ± standard deviation (SD), whereas non-normally distributed variables are reported as median (interquartile range). Categorical variables are expressed as frequencies and percentages (%).

Demographic characteristics were compared between the migraine and control groups using the independent-samples *t*-test or the Mann–Whitney *U* test, as appropriate based on data distribution. Categorical variables were analyzed using the chi-square test or Fisher’s exact test. Clinical and demographic characteristics of the study population were summarized using descriptive statistics. Because the study population was restricted to a relatively narrow developmental age range (12–17 years) and no statistically significant between-group differences were identified in age or sex distribution, additional age- or sex-adjusted ANCOVA analyses were not performed.

For the primary morphometric analysis, measurements from 135 cortical, subcortical, cerebellar, and CSF-related anatomical regions obtained from the vol2Brain platform were compared between the migraine and control groups. Secondary analyses included cerebellar subregional volumes, cerebellar gray matter volumes, cerebellar cortical thickness measurements, and thalamic nuclei volumes derived from the integrated FreeSurfer/3D Slicer workflow. To quantify the magnitude of group differences, Hedges’ g effect sizes and corresponding 95% confidence intervals (CI) were calculated.

To reduce the risk of type I error associated with multiple anatomical region comparisons, the Benjamini–Hochberg false discovery rate (FDR) correction was applied. FDR correction was performed separately for distinct methodological analysis families. Accordingly, independent FDR correction procedures were applied to whole-brain morphometric analyses, cerebellar subregional volume analyses, cerebellar gray matter analyses, cerebellar cortical thickness analyses, and thalamic nuclei analyses. Adjusted q values < 0.05 were considered statistically significant. Both raw *p* values and FDR-adjusted q values were reported. To further evaluate the potential influence of residual confounding, all morphometric parameters that remained statistically significant after Benjamini–Hochberg false discovery rate correction were reanalyzed using analysis of covariance (ANCOVA). Age, sex, and body mass index were included as covariates in all models, whereas total intracranial volume was additionally included as a covariate for volumetric measurements. The resulting ANCOVA-derived *p* values were subsequently adjusted using the Benjamini–Hochberg false discovery rate procedure within each corresponding analysis family. Morphometric findings that remained statistically significant after both covariate adjustment and FDR correction were considered to demonstrate robustness against potential demographic and anthropometric confounding. Because of the retrospective nature of the study, an a priori sample size calculation was not performed. Instead, a post hoc power analysis was conducted based on the final cohort of 74 patients with migraine without aura and 70 healthy controls. At a two-sided significance level of α = 0.05, the sample size provided approximately 85% power to detect a moderate effect size (d = 0.50) and approximately 90% power to detect an effect size of d = 0.54.

Additional confirmatory analyses were performed for volumetric parameters that remained statistically significant after FDR correction. To evaluate the potential influence of interindividual differences in head size, only volumetric measures that retained significance following FDR adjustment were normalized for total intracranial volume (TIV). TIV correction was performed using a proportional scaling approach according to the formula: regional volume/individual TIV × mean cohort TIV. The resulting TIV-adjusted volumetric measurements were subsequently reanalyzed and compared between the migraine and control groups. Findings that remained significant after TIV adjustment were reported separately.

To assess the reproducibility of the visual quality control process for automated segmentation outputs, an interobserver reliability analysis was conducted in a randomly selected subgroup comprising 15% of the study population. In this analysis, segmentation results were independently re-evaluated by a second experienced neuroradiologist who was blinded to clinical group allocation. Interobserver agreement was quantified using the intraclass correlation coefficient (ICC).

For all analyses, a two-sided *p* value < 0.05 was considered statistically significant. For morphometric analyses involving multiple comparisons, statistical significance was determined based on an FDR-adjusted q value < 0.05.

## 3. Results

### 3.1. Study Population and Demographic Characteristics

The final study cohort consisted of 74 patients with migraine without aura and 70 control subjects with structurally normal cranial MRI findings. No statistically significant differences were observed between the groups with respect to age (14.8 ± 1.6 years vs. 14.3 ± 1.5 years, *p*-value = 0.071), sex distribution (*p*-value = 0.337), or body mass index (*p*-value = 0.249).

In the migraine group, the mean disease duration was 2.6 ± 1.4 years, and the mean age at migraine onset was 12.2 ± 1.8 years. The mean attack frequency was 4.1 ± 2.3 attacks per month, with a mean attack duration of 13.6 ± 8.4 h. The most common associated symptoms were unilateral headache (66.2%), phonophobia (60.8%), and nausea/vomiting (56.8%). Pulsating headache characteristics were reported in 68.9% of patients, whereas a unilateral pain pattern was present in 45.9% of cases.

Detailed demographic and clinical characteristics of the study population are presented in Table 2.

### 3.2. Magnetic Resonance Imaging Quality Control and Segmentation Reliability

Following the application of the predefined MRI quality control and segmentation validation criteria, a total of 61 MRI datasets were excluded prior to volumetric analysis. The primary reasons for exclusion included image quality–related technical issues, inadequate anatomical coverage, and failure of segmentation validation. Detailed information regarding the quality control process and exclusion criteria is presented in Figure 2.

After completion of the multistep quality control and anatomical verification procedures, all imaging datasets from the final study cohort, comprising 74 patients with migraine without aura and 70 control subjects, met the predefined quality requirements for volumetric analysis. Interobserver reliability analysis was performed in a randomly selected subgroup representing 15% of the study population. Volumetric measurements demonstrated excellent reproducibility, with an intraclass correlation coefficient (ICC) of 0.96 (95% CI: 0.92–0.98).

### 3.3. Whole-Brain Volumetric Findings

Whole-brain morphometric analysis revealed statistically significant differences between the migraine and control groups in bilateral amygdala volumes, right thalamic volume, and cortical thickness of the right anterior insula, left anterior cingulate gyrus, and precuneus. All of these findings remained significant after Benjamini–Hochberg FDR correction and were associated with small-to-moderate effect sizes (Hedges’ g = 0.39–0.61) (Table 3).

Compared with the control group, patients with migraine without aura exhibited lower bilateral amygdala and right thalamic volumes, as well as reduced cortical thickness in the right anterior insula, left anterior cingulate gyrus, and precuneus. The largest effect sizes were observed in the right anterior insula (Hedges’ g = 0.61), left anterior cingulate gyrus (Hedges’ g = 0.59), and bilateral amygdala volumes (Hedges’ g = 0.56–0.58). No additional anatomical regions demonstrated statistically significant differences following FDR correction.

### 3.4. Cerebellar Subregional Analysis

Cerebellar subregional volumetric analysis demonstrated bilateral volume reductions in Lobule VI, and these differences remained statistically significant after Benjamini–Hochberg FDR correction (Hedges’ g = 0.43–0.46) (Table 4). Similarly, gray matter volume analysis revealed significantly lower bilateral gray matter volumes of Lobule VI in the migraine group, with moderate effect sizes (Hedges’ g = 0.60–0.64), and these findings remained significant following FDR correction (Table 5).

In the cortical thickness analysis, significant cortical thinning was identified in the right Crus I and right Crus II in patients with migraine without aura. These differences also remained statistically significant after correction for multiple comparisons and were associated with moderate effect sizes (Hedges’ g = 0.61–0.66) (Table 6). No statistically significant differences were observed in the remaining cerebellar lobules with respect to volume, gray matter volume, or cortical thickness measurements after FDR correction.

### 3.5. Thalamic Nuclei Findings

Thalamic nuclei volumetric analysis demonstrated significantly lower bilateral mediodorsal nucleus (MN) and pulvinar nucleus (PN) volumes, as well as lower right ventral anterior nucleus (VAN) and right ventral posterolateral nucleus (VPLN) volumes, in the migraine group compared with the control group. These differences remained statistically significant after Benjamini–Hochberg FDR correction and were associated with moderate to moderately large effect sizes (Hedges’ g = 0.53–0.68) (Table 7). The largest effect sizes were observed in the bilateral pulvinar nucleus and mediodorsal nucleus. No statistically significant volumetric differences were identified in the remaining thalamic nuclei following FDR correction.

### 3.6. Total Intracranial Volume-Normalized Volumetric Findings

In the confirmatory analyses performed on volumetric parameters that remained statistically significant after Benjamini–Hochberg FDR correction in the primary analyses, all significant findings identified in the whole-brain morphometric analyses, cerebellar subregional analyses, and thalamic nuclei analyses remained statistically significant after adjustment for total intracranial volume (TIV). The corresponding effect sizes remained largely unchanged following TIV normalization (Hedges’ g = 0.42–0.66), supporting the robustness of the observed morphometric differences and indicating that the findings were not primarily driven by interindividual variations in intracranial volume (Table 8).

To further assess the potential influence of demographic and anthropometric confounding variables, all morphometric parameters that remained statistically significant after FDR correction were reanalyzed using ANCOVA. Adjustment for age, sex, and BMI, with additional adjustment for TIV for volumetric measurements, did not materially alter the statistical significance, direction, or overall pattern of the observed morphometric findings. All previously significant morphometric findings remained statistically significant following covariate adjustment. The complete ANCOVA-adjusted results are provided in Supplementary Material S4.

To facilitate interpretation of the principal findings and provide an integrated overview of the morphometric alterations identified across the different analytical levels, a summary of all parameters that remained statistically significant after Benjamini–Hochberg false discovery rate correction is presented in Table 9.

## 4. Discussion

In the present study, a multiplatform neuroimaging approach integrating whole-brain volumetry, cerebellar subregional analyses, and thalamic nuclei assessment identified morphometric alterations involving cortical, limbic, thalamic, and cerebellar structures in adolescents aged 12–17 years with migraine without aura. The fact that these findings were not confined to a single anatomical region but were observed across multiple functionally interconnected cortical and subcortical structures suggests that migraine may not be adequately characterized as a localized brain disorder. In particular, the concurrent structural alterations identified within anatomical systems known to participate in pain modulation, sensory integration, emotional processing, salience attribution, and cortical–subcortical information transfer support the concept that migraine may be associated with a distributed neuroanatomical pattern within the developing brain. Accordingly, the present findings are broadly consistent with contemporary neurobiological models proposing that migraine is not a disorder arising from a single structural focus but rather is associated with morphometric alterations involving anatomical regions implicated in pain processing, emotional regulation, sensory integration, and cortical–subcortical communication [41,42,43,44,45]. Notably, the findings related to the thalamic nuclei and cerebellar subregions represent some of the most distinctive and potentially novel contributions of the present study to the existing literature.

An important consideration when interpreting the present findings is that the study focused on a neurodevelopmentally more homogeneous pediatric cohort aged 12–17 years, thereby minimizing the pronounced developmental variability characteristic of early childhood. This age range represents a critical neurodevelopmental period during which cortical maturation, white matter development, and the refinement of anatomical organization between cortical and subcortical systems continue to progress, although with less variability than in younger children. Synaptic pruning, experience-dependent neuroplasticity, myelination, and the maturation of distinct cortical and subcortical networks remain active during this stage and exert substantial influences on brain structural organization. Consequently, migraine-related morphometric alterations may reflect not only ongoing disease processes but also the dynamic developmental mechanisms of the maturing brain. Neuroimaging findings reported in adult migraine populations may be influenced by long-term disease burden, repeated pain exposure, environmental factors, and age-related neuroanatomical changes. In contrast, findings obtained from a neurodevelopmentally more homogeneous adolescent cohort may provide insight into earlier stages of the structural organization patterns associated with migraine. Therefore, direct extrapolation of results from adult cohorts to pediatric populations may not be appropriate, and age-specific neuroimaging studies are needed to improve our understanding of the neurodevelopmental biology of migraine [46,47,48,49].

In the present study, lower bilateral amygdala volumes and reduced cortical thickness of the left anterior cingulate gyrus were observed in the migraine group, suggesting that migraine may be associated with morphometric alterations in limbic structures involved in emotional processing and the affective dimensions of pain. The limbic system comprises a network of neuroanatomical structures implicated in the evaluation of the emotional aspects of pain, the formation of pain expectancy, the maintenance of learned pain-related responses, and the modulation of pain memory. As a key component of this system, the amygdala has been linked to the affective evaluation of nociceptive stimuli and central sensitization processes, whereas the anterior cingulate cortex is considered one of the principal regions involved in the emotional dimension of pain, attentional processing, and the regulation of behavioral responses [5,9,16]. Several neuroimaging studies conducted in adult migraine populations have reported findings supporting the involvement of these structures in migraine pathophysiology. Schwedt et al. identified altered functional responses in pain-processing regions, including the anterior cingulate cortex and insula, in patients with episodic migraine and suggested that these regions may play important roles in migraine-related processes [16]. Similarly, a systematic review by Schramm et al. emphasized the presence of functional alterations within large-scale brain systems encompassing the limbic network, salience-processing regions, and pain-related structures in individuals with migraine [9]. In a more recent study, Kosuge et al. reported reduced amygdala volume, particularly in the right amygdala, in patients with migraine and suggested that this finding may reflect migraine-related alterations within the limbic system [50]. Likewise, Ou et al. demonstrated both structural and functional abnormalities in subregions of the anterior cingulate cortex in individuals with migraine and proposed that these changes may be associated with altered pain-processing mechanisms [51]. Nevertheless, findings regarding the amygdala and anterior cingulate cortex remain inconsistent across the literature. While some studies have reported volume loss or cortical thinning, others have described increased volumes or failed to identify significant morphometric differences [5,9,50,51]. Evidence in pediatric migraine populations is even more limited. Bell et al. identified age-related functional alterations in pediatric migraine patients and emphasized the importance of interpreting migraine within a neurodevelopmental framework [6]. Guarnera et al. reported differences in cortical thickness and gyrification across several cortical regions in pediatric patients with migraine without aura [13]. Furthermore, a review by Webb et al. highlighted that neuroimaging findings in pediatric migraine do not fully mirror those reported in adult populations and should therefore be interpreted in the context of ongoing neurodevelopment [14]. This heterogeneity may be attributable to differences in age distribution, migraine subtype, disease duration, imaging methodology, sample size, and segmentation techniques. In addition, the continued neurodevelopment and experience-dependent plasticity of limbic structures such as the amygdala and anterior cingulate cortex during childhood and adolescence may contribute to variability across studies. Within this context, the identification of both bilateral amygdala volume reductions and anterior cingulate cortical alterations in our neurodevelopmentally more homogeneous cohort of adolescents aged 12–17 years suggests that migraine may be associated not only with the sensory dimension of pain but also with morphometric differences in limbic structures involved in its emotional and cognitive components. Overall, our findings are broadly consistent with the existing literature supporting a potential role of the limbic system in migraine pathophysiology. However, prospective longitudinal studies are required to clarify the directionality and biological significance of these associations.

An additional framework through which the present findings may be interpreted is central sensitization, a neurobiological process characterized by enhanced responsiveness of nociceptive pathways within the central nervous system following repeated or sustained nociceptive input [7,10,11]. Contemporary models of migraine propose that recurrent activation of the trigeminovascular system may contribute not only to transient pain generation but also to longer-term alterations in neural systems involved in sensory processing, pain modulation, and salience attribution [7,42,52]. Within this context, the concurrent morphometric alterations identified in the anterior insula, anterior cingulate cortex, amygdala, and thalamus are of particular interest because these regions occupy central positions within anatomical networks implicated in the processing, evaluation, integration, and modulation of nociceptive information. The anterior insula and anterior cingulate cortex constitute major components of the salience network and are involved in interoceptive awareness, attentional allocation to biologically relevant stimuli, cognitive–emotional appraisal of pain, and behavioral response generation [9,42,51]. The amygdala contributes to affective pain processing, pain-related learning, emotional regulation, and descending modulatory mechanisms, whereas the thalamus functions as a critical relay and filtering structure responsible for the integration and transmission of nociceptive and sensory information to distributed cortical systems [7,18,52]. Consequently, the co-occurrence of structural alterations across these anatomically and functionally interconnected regions may be compatible with a neurobiological framework in which repeated migraine-related nociceptive activation is associated with activity-dependent reorganization within cortical, limbic, and thalamocortical networks. Nevertheless, the present findings should not be interpreted as direct evidence of central sensitization. Because the current investigation was designed as a structural morphometric study and did not include quantitative sensory testing, electrophysiological assessments, functional connectivity analyses, or longitudinal clinical measurements, direct evaluation of sensitization-related mechanisms was beyond its scope. Therefore, central sensitization is best regarded as a biologically plausible interpretive framework that may help contextualize the observed morphometric alterations rather than as a mechanistic conclusion directly established by the present data.

The reduced cortical thickness observed in the right anterior insula and left anterior cingulate gyrus in our study suggests a potential association between migraine and morphometric alterations in anatomical regions involved in salience processing, attentional control, and the cognitive–emotional dimensions of pain. Salience processing refers to the neurobiological mechanisms responsible for identifying behaviorally relevant internal and external stimuli, prioritizing sensory inputs, and directing attentional resources toward salient information. Among the principal structures implicated in these processes, the anterior insula and anterior cingulate cortex are thought to contribute to the detection of painful stimuli, the cognitive and emotional appraisal of pain, and the generation of appropriate behavioral responses [9,51]. Numerous voxel-based morphometry and cortical thickness studies in migraine populations have reported structural alterations involving these regions. Schmitz et al. identified gray matter changes in pain-processing regions, including the insula and cingulate cortex, in patients with migraine and suggested that these findings may reflect central neurobiological mechanisms associated with the disorder [53]. Similarly, Rocca et al. reported morphometric alterations involving the anterior cingulate cortex, insula, and related cortical regions in migraine patients [54]. Reviews by Messina et al. have highlighted the anterior insula and anterior cingulate cortex as among the most consistently affected regions in migraine neuroimaging studies, emphasizing their roles within anatomical systems involved in salience attribution and pain processing. Likewise, Chong et al., in their review of cortical morphometry studies, concluded that structural alterations involving the insular and cingulate cortices may be associated with migraine-related neurobiological processes [55]. More recently, Ou et al. demonstrated both structural and functional abnormalities in anterior cingulate cortex subregions in patients with migraine and suggested that these alterations may be linked to pain-processing mechanisms [51]. However, the direction of the reported findings remains inconsistent across studies. While some investigations have demonstrated cortical thinning, others have reported increased cortical thickness or no significant morphometric differences [5,9,51,53,54,55]. Evidence from pediatric migraine populations is comparatively limited. Bell et al. identified age-dependent functional alterations in pediatric migraine patients and emphasized the importance of interpreting migraine within a neurodevelopmental framework [6]. Guarnera et al. reported differences in cortical thickness and gyrification across several cortical regions in pediatric patients with migraine without aura [13]. Furthermore, Webb et al. highlighted in their review that neuroimaging findings in pediatric migraine do not fully correspond to those reported in adult cohorts and should therefore be interpreted in the context of ongoing neurodevelopment [14]. This heterogeneity may reflect differences in study populations, migraine subtypes, disease duration, attack frequency, imaging methodologies, and analytical strategies. The morphometric alterations observed in the anterior insula and anterior cingulate cortex in our cohort suggest that migraine may be associated not only with the sensory aspects of pain but also with structural differences in regions involved in salience attribution, cognitive appraisal, and behavioral responses to pain. Importantly, when considered alongside the limbic alterations identified in the present study, these findings suggest that migraine-related morphometric differences may involve anatomically interconnected systems participating in salience attribution, cognitive appraisal, and pain modulation, highlighting the potential interaction between sensory and affective dimensions of migraine.

The reduced cortical thickness of the precuneus observed in the migraine group suggests that migraine may be associated not only with pain-processing mechanisms but also with morphometric alterations in brain regions involved in internally directed cognitive functions and self-referential processing. The precuneus is considered a core anatomical component of the default mode network (DMN) and has been implicated in self-awareness, autobiographical memory, internally generated thought processes, and cognitive activities that occur independently of external stimuli. In addition, the precuneus is thought to maintain extensive connections with cortical systems involved in cognitive control, attentional regulation, and internally directed cognition. Functional neuroimaging studies of migraine have also reported alterations involving the DMN. Zhang et al. demonstrated increased functional connectivity and enhanced regional homogeneity within the precuneus/posterior cingulate cortex in patients with migraine without aura and proposed that this region may represent an important hub for pain sensitivity and DMN-related processes [56]. Similarly, Tessitore et al. reported alterations in DMN functional connectivity in patients with migraine without aura and suggested that these findings may reflect migraine-related functional abnormalities [57]. Coppola et al. further demonstrated that connectivity between the DMN and insula during acute migraine attacks may be associated with pain severity [58]. More recently, Schramm et al., in a systematic review, highlighted recurring connectivity alterations across multiple functional brain systems, including the DMN, in individuals with migraine [9]. In contrast to the relatively consistent functional findings, structural MRI results involving the precuneus have been less uniform. Considerable variability exists among studies evaluating regional volumes and cortical thickness, and reported findings have not been entirely consistent. Such discrepancies may be attributable to differences in sample characteristics, migraine subtypes, disease duration, imaging methodologies, and analytical approaches. The precuneal cortical thinning identified in the present study suggests that migraine may not be exclusively related to pain perception but may also involve morphometric alterations in regions associated with self-referential awareness and internally directed cognitive processes. Importantly, when interpreted alongside the limbic and salience-related alterations identified in the present study, the precuneal findings suggest that migraine-associated morphometric differences may extend beyond pain-processing systems and involve regions contributing to internally directed cognitive processes and large-scale network organization.

The reduced right thalamic volume observed in the migraine group is consistent with the proposed central role of the thalamus in migraine pathophysiology. The thalamus serves as a major relay station for nociceptive information originating from the trigeminovascular system and plays a critical role in the filtering, integration, and modulation of sensory inputs before their transmission to cortical regions. Goadsby et al. characterized migraine as a disorder of sensory processing and emphasized that pain signals arising from the trigeminovascular system are relayed to cortical areas through thalamic centers [7]. Similarly, Akerman et al. highlighted the importance of diencephalic structures, particularly the thalamus, in the processing of pain-related information in migraine [18]. In a more recent review, Younis et al. suggested that the thalamus may contribute not only to pain transmission but also to several clinical manifestations of migraine, including photophobia, phonophobia, cutaneous allodynia, and central sensitization [52]. Functional neuroimaging studies further support this concept. Martinelli et al. demonstrated alterations in thalamocortical connectivity during experimentally induced migraine attacks and proposed that the thalamus participates in dynamic neurophysiological processes associated with migraine [59]. However, findings from structural MRI studies have been less consistent. While some investigations have reported volumetric or microstructural thalamic alterations, others have failed to identify significant differences [52,59,60,61]. This heterogeneity may be related to variations in migraine subtype, aura status, disease duration, attack frequency, and imaging methodologies. Within this context, the reduced thalamic volume identified in our cohort suggests that migraine may be associated with structural alterations in anatomical systems involved in the processing, filtering, and cortical transmission of sensory information within the developing brain. Nevertheless, the present study was not designed to evaluate functional connectivity or network organization, and therefore no direct conclusions can be drawn regarding the potential impact of the observed morphometric differences on thalamocortical communication. Furthermore, owing to the cross-sectional nature of the study, the directionality of this association cannot be determined.

Beyond central sensitization, abnormal habituation and altered sensory gain control constitute additional neurophysiological frameworks that may help contextualize the present findings [11]. Habituation refers to the progressive attenuation of neural responses during repetitive sensory stimulation and is generally considered an adaptive mechanism that prevents excessive allocation of neural resources to recurring sensory inputs. However, accumulating electrophysiological evidence suggests that individuals with migraine frequently exhibit deficient habituation across multiple sensory modalities, characterized by persistent or exaggerated responses to repetitive stimulation [11]. This phenomenon has been interpreted as reflecting abnormalities in sensory gating, cortical excitability regulation, and thalamocortical processing. Within this framework, the thalamus is of particular relevance because it serves as a major hub for the filtering, integration, and transmission of sensory information to distributed cortical networks [7,52]. Accordingly, the nucleus-specific thalamic alterations identified in the present study may be biologically meaningful in the context of abnormal sensory processing mechanisms that have been repeatedly implicated in migraine. The bilateral involvement of the pulvinar nucleus is especially noteworthy. The pulvinar participates in visual attention, multisensory integration, attentional selection, and sensory filtering processes and has been proposed as an important regulator of sensory information flow across large-scale brain networks [52,60]. Given that migraine is frequently associated with photophobia, phonophobia, sensory hypersensitivity, and enhanced responsiveness to environmental stimuli, the pulvinar alterations observed in the present study may be compatible with contemporary models proposing dysregulated sensory gain control and impaired filtering of sensory information. Nevertheless, because the present investigation did not include electrophysiological assessments, sensory testing paradigms, or functional neuroimaging analyses, no direct conclusions can be drawn regarding habituation deficits or sensory gain abnormalities. Therefore, these mechanisms should be regarded as biologically plausible interpretive frameworks that may help contextualize the observed morphometric alterations rather than as direct mechanistic explanations established by the current data.

Beyond the global thalamic findings, the separate evaluation of individual thalamic nuclei represents one of the most distinctive aspects of the present study. Thalamic nuclei analysis demonstrated significantly lower bilateral mediodorsal nucleus and pulvinar nucleus volumes, as well as reduced right ventral anterior nucleus and right ventral posterolateral nucleus volumes, in the migraine group compared with the control group. Importantly, all of these differences remained statistically significant after Benjamini–Hochberg FDR correction, reducing the likelihood that the observed findings can be attributed to chance effects arising from multiple comparisons. These results suggest that migraine-related morphometric alterations may not be uniformly distributed throughout the thalamus but instead may preferentially involve specific thalamic subregions. Such a pattern supports the notion that nucleus-level analyses may provide additional neuroanatomical information beyond that obtained from global thalamic volumetric measurements and may reveal region-specific structural alterations that would otherwise remain undetected. The thalamus is not a functionally homogeneous structure; rather, its individual nuclei exhibit distinct connectivity patterns with specific cortical and subcortical regions. The mediodorsal nucleus is primarily associated with emotional and cognitive processes through its extensive connections with prefrontal and limbic structures, whereas the ventral nuclei are principally involved in somatosensory information processing and transmission. The pulvinar nucleus has been implicated in visual attention, multisensory integration, and the processing of sensory information. Consequently, assessment of total thalamic volume alone may overlook nucleus-specific alterations potentially associated with migraine. Younis et al. emphasized that understanding migraine pathophysiology requires evaluation of the thalamus through its functional subcomponents rather than as a single anatomical entity [52]. Supporting this concept, Shin et al. reported volumetric differences in individual thalamic nuclei in adults with migraine and suggested that specific thalamic subregions may be selectively involved in the disorder [60]. In contrast, Giardina et al. found no significant macrostructural differences in either total thalamic volume or thalamic subregions in patients with episodic migraine without aura examined during the interictal period [61]. These discrepant findings suggest that thalamic nuclei measurements may be influenced by sample characteristics, timing of imaging, disease-related factors, and analytical methodologies. In the present study, the most prominent and bilaterally distributed findings involved the mediodorsal and pulvinar nuclei. The mediodorsal nucleus is closely connected to the prefrontal cortex and limbic structures and has been associated with emotional regulation, cognitive appraisal, and behavioral response processes. In this context, the bilateral reduction in mediodorsal nucleus volume is particularly noteworthy when considered alongside the morphometric alterations identified in the amygdala and anterior cingulate cortex. Although functional interactions among these regions were not assessed in the present study, the co-occurrence of structural alterations in anatomical regions implicated in similar emotional and cognitive functions represents an intriguing observation. Similarly, bilateral pulvinar nucleus volume reduction constitutes one of the most notable findings of the study. The pulvinar is involved in visual attention, multisensory integration, and sensory filtering. Given the clinical manifestations of migraine, including photophobia, phonophobia, and sensory hypersensitivity, the morphometric alterations observed at the level of the pulvinar may be biologically meaningful. Moreover, the effect sizes observed for the pulvinar nucleus were among the largest in the present study, suggesting that this region may be particularly relevant to migraine-associated structural alterations. The reductions observed in the right ventral anterior nucleus and right ventral posterolateral nucleus should be interpreted as more limited and lateralized findings. The ventral posterolateral nucleus is recognized as a principal thalamic relay nucleus involved in somatosensory information transmission, whereas the ventral anterior nucleus contributes to functions associated with motor and premotor cortical networks. The biological significance of these findings remains uncertain and requires confirmation in larger cohorts. Notably, studies investigating thalamic nuclei at the subnuclear level in pediatric migraine populations remain exceedingly scarce. Existing pediatric neuroimaging literature has focused predominantly on global brain volumetry, cortical thickness measurements, or functional neuroimaging approaches, whereas detailed evaluation of thalamic subregions has largely been overlooked [4,6,13,14]. Therefore, by separately examining individual thalamic nuclei in a neurodevelopmentally more homogeneous cohort of adolescents aged 12–17 years, the present study addresses an important gap in the literature. Overall, our findings suggest that migraine may be associated not only with global thalamic alterations but also with selective morphometric differences involving specific thalamic subregions. However, because the present study was not designed to evaluate functional connectivity, no direct conclusions can be drawn regarding whether these structural alterations are associated with functional interactions among thalamic nuclei or within broader thalamocortical networks.

The observation of reduced bilateral Lobule VI volumes and gray matter volumes in the migraine group is consistent with the growing body of literature suggesting a potential role for the cerebellum in migraine pathophysiology. Traditionally, the cerebellum has been primarily associated with motor coordination and balance; however, accumulating anatomical and functional evidence indicates that it may also contribute to pain modulation, sensory integration, autonomic regulation, and higher cognitive processes [12,15,17]. Noseda et al. proposed that the cerebellum constitutes an important component of the neuroanatomical systems potentially involved in the generation of migraine symptoms [12]. Similarly, Rudolph et al. emphasized that cerebellar function extends beyond motor control and includes participation in cognitive and affective processes [15]. In the context of migraine, Matoso et al. reported structural alterations involving cerebellar regions in patients with episodic migraine and highlighted the potential importance of the cerebellum in migraine biology [44]. Lobule VI is generally regarded as a transitional zone between sensorimotor and cognitive cerebellar territories and has been associated with somatosensory information processing, pain modulation, and multisensory integration [17]. Therefore, the bilateral reductions in both total volume and gray matter volume of Lobule VI identified in our study suggest that migraine-related morphometric alterations may involve not only cortical and thalamic structures but also cerebellar subregions implicated in sensory processing and modulation. The bilateral nature of these findings is particularly noteworthy, as it argues against a chance lateralization effect and may instead reflect a more widespread neuroanatomical pattern. Nevertheless, because of the cross-sectional design of the present study, it is not possible to determine whether these morphometric differences represent predisposing factors for migraine, consequences of the disorder, or adaptive changes that develop over the course of the disease.

Beyond the evidence implicating the cerebellum in migraine, the relationship between the posterior cerebellum and higher cognitive functions is also of particular interest. In the present study, reduced cortical thickness was identified in the right Crus I and right Crus II. Contemporary cerebellar neuroscience research has demonstrated that the posterior cerebellum, particularly the Crus I and Crus II regions, is involved in executive functions, attentional control, working memory, and higher-order cognitive processes [15,17]. Functional neuroimaging studies have further shown that these regions constitute important components of functional systems associated with the frontoparietal network, executive control network, and DMN. Guell and Schmahmann demonstrated that Crus I and Crus II contribute to cognitive processing through their extensive anatomical and functional connections with prefrontal and parietal cortical regions [17]. Likewise, Rudolph et al., in their review of cerebellar cognitive-affective functions, emphasized the close involvement of posterior cerebellar regions in higher cognitive processes [15]. Although studies specifically investigating cerebellar cognitive systems in migraine remain relatively limited, Zhou et al. reported alterations in the dynamic resting-state functional organization of patients with migraine and suggested that these changes may extend beyond pain-processing mechanisms alone [8]. Similarly, the systematic review by Schramm et al. highlighted that migraine is associated with abnormalities across several functional systems, including the DMN and executive control networks [9]. However, the present study was not designed to evaluate functional connectivity, and therefore no direct conclusions can be drawn regarding whether the observed structural alterations are reflected in these functional systems. Within this context, the cortical thinning observed in Crus I and Crus II suggests that migraine-related morphometric alterations may not be restricted to regions traditionally associated with pain processing but may also involve cerebellar subregions implicated in attention, cognitive control, and higher-order cognitive functions. Importantly, when these findings are considered together with the morphometric alterations identified in the precuneus, anterior insula, anterior cingulate cortex, thalamus, and specific thalamic nuclei, they suggest that cerebellar structural differences may coexist with alterations in regions implicated in sensory integration, attentional regulation, and higher-order cognitive processing.

An important consideration in the interpretation of the present findings concerns the biological significance and temporal directionality of the observed morphometric alterations. Because the current study employed a cross-sectional design, it is not possible to determine whether the identified structural differences represent consequences of migraine, pre-existing neuroanatomical characteristics associated with disease susceptibility, or adaptive neuroplastic responses that develop over time. Consequently, the observed alterations should not be interpreted as unequivocal markers of disease-related structural injury. Several non-mutually exclusive explanatory frameworks may be considered. First, repeated activation of trigeminovascular pathways, recurrent nociceptive processing, sensory hypersensitivity, and long-term exposure to migraine-related neurobiological mechanisms may contribute to activity-dependent structural remodeling within cortical, limbic, thalamic, and cerebellar systems [5,7,9]. Within this framework, the observed morphometric differences could represent consequences of recurrent migraine-related physiological processes. Alternatively, some structural characteristics may precede the clinical manifestation of migraine and constitute neuroanatomical traits that influence susceptibility to altered sensory processing, pain modulation, or migraine development itself. A third possibility is that the identified alterations reflect adaptive or maladaptive neuroplastic responses occurring in response to recurrent migraine experiences, thereby representing compensatory reorganization rather than direct pathological injury. This issue may be particularly relevant in pediatric populations because adolescence is characterized by ongoing cortical maturation, synaptic refinement, myelination, and experience-dependent neuroplasticity [23,28,46,48,49]. Consequently, the structural differences observed in the present cohort may reflect dynamic interactions between migraine-related biological mechanisms and neurodevelopmental processes rather than fixed anatomical abnormalities. Future longitudinal studies incorporating repeated clinical and neuroimaging assessments will be necessary to determine whether these morphometric alterations precede migraine onset, emerge during disease progression, or evolve as adaptive neuroplastic responses over time.

Overall, the morphometric alterations identified in the present study suggest that migraine may be associated with a distributed neuroanatomical pattern involving multiple cortical, subcortical, and cerebellar structures rather than a single localized anatomical region. At the cortical level, the alterations observed in the anterior insula, anterior cingulate cortex, and precuneus involve regions implicated in pain processing, attentional regulation, and cognitive appraisal. At the limbic level, the volumetric reductions identified in the amygdala may reflect processes related to the emotional dimensions of pain. In addition, the findings involving the thalamus and specific thalamic nuclei suggest that morphometric alterations within anatomical systems responsible for the processing, filtering, and cortical transmission of sensory information may also be associated with migraine. At the cerebellar level, the structural differences identified in Lobule VI, Crus I, and Crus II indicate that migraine-related morphometric alterations may not be confined to pain-processing mechanisms alone but may also involve cerebellar subregions associated with sensory integration, cognitive control, and higher-order cognitive functions. Taken together, these findings support the conceptualization of migraine as a condition associated with distributed morphometric alterations involving cortical, limbic, thalamic, and cerebellar structures [7,9,12,15,52]. Among the most notable and distinctive findings of the present study were the bilateral volume reductions observed in the mediodorsal and pulvinar nuclei, together with the bilateral alterations identified in Lobule VI and the cortical thinning observed in the right Crus I and Crus II. Nevertheless, the observed effect sizes generally ranged from small to moderate, suggesting that the findings reflect subtle morphometric differences associated with migraine rather than pronounced structural abnormalities.

The present study has several notable strengths. First, to minimize neurodevelopmental variability associated with early childhood, the analysis was restricted to a neurodevelopmentally more homogeneous cohort of adolescents aged 12–17 years. In addition, only patients with migraine without aura were included, thereby reducing clinical heterogeneity within the study population. From a statistical perspective, Benjamini–Hochberg FDR correction was applied to account for multiple comparisons, and all volumetric measurements were normalized to total intracranial volume in an effort to reduce both false-positive findings and the influence of potential confounding factors. Importantly, the persistence of significant findings after total intracranial volume normalization suggests that the observed differences cannot be explained solely by interindividual variation in head size. Another important strength is the implementation of a multiplatform neuroimaging workflow that combined vol2Brain for whole-brain volumetric assessment, FreeSurfer for thalamic nuclei segmentation, and 3D Slicer for segmentation verification and quality control. This integrated approach enabled the complementary strengths of different software platforms to be leveraged, facilitating a comprehensive neuroanatomical evaluation. Furthermore, segmentation accuracy was systematically assessed through detailed quality control procedures, and excellent interobserver agreement was achieved (ICC = 0.96), supporting the reproducibility of the volumetric measurements. Collectively, these methodological features enhance both the robustness of the study design and the reliability of the reported findings.

Several limitations of the present study should be acknowledged. First, the retrospective single-center design does not completely eliminate the possibility of selection bias. In addition, because of the cross-sectional nature of the study, neither the temporal evolution nor the causal direction of the observed structural alterations could be determined. Another important limitation is the lack of available data regarding pubertal developmental status. Because pubertal information was not retrospectively accessible, the potential influence of pubertal maturation on the observed morphometric differences could not be evaluated. Although the study was restricted to individuals aged 12–17 years to reduce neurodevelopmental variability associated with early childhood and the migraine and control groups were comparable with respect to age and sex distribution, pubertal maturation is known to influence brain development substantially. Therefore, the inability to assess Tanner stage or hormonal markers should be considered a significant limitation. Moreover, chronological age may not fully reflect biological maturation status, and adolescents of similar age may exhibit substantially different stages of pubertal development. Although age and sex distributions were comparable between groups and the study population was restricted to a relatively narrow developmental age range, residual neurodevelopmental variability cannot be completely excluded. The composition of the control group also warrants careful consideration. The control cohort did not consist of completely asymptomatic healthy volunteers but rather of individuals who underwent cranial MRI for clinical indications. Nevertheless, all control participants underwent comprehensive neurological evaluation, demonstrated structurally normal MRI findings, and had no history of neurological, neurodevelopmental, psychiatric, or systemic disorders that could influence brain morphology. Furthermore, all control MRI examinations were independently re-evaluated by an experienced neuroradiologist to minimize the possibility of overlooked structural abnormalities. Consequently, the control group was carefully selected to enhance its suitability for neuroanatomical comparisons and may be regarded as a well-characterized clinical control cohort. However, the use of hospital-based controls rather than community-based healthy vol unteers represents an inherent methodological limitation and raises the possibility of residual confounding. Although the neurological complaints prompting MRI referral were transient, non-focal, and ultimately not associated with identifiable structural abnormalities, it cannot be completely excluded that factors leading to MRI referral may have influenced brain morphology. Therefore, this issue should be considered when interpreting the observed morphometric differences between groups. Importantly, the use of community-based healthy pediatric controls was not feasible within the context of the present retrospective cross-sectional study. Because MRI examinations are not routinely performed in asymptomatic children without a clinical indication, retrospective imaging archives rarely contain high-quality three-dimensional MRI datasets from completely healthy pediatric volunteers. Consequently, the identification of a sufficiently large, age-matched cohort of neurologically normal children with standardized volumetric MRI acquisitions is often not possible in retrospective pediatric neuroimaging studies. Furthermore, the prospective acquisition of research-dedicated MRI examinations in healthy children presents substantial ethical, regulatory, and practical challenges. Institutional Review Boards generally apply strict scrutiny to non-clinically indicated MRI studies in pediatric populations, particularly when prolonged imaging protocols, participant compliance, motion-related image degradation, and, in younger age groups, concerns regarding the potential need for sedation must be considered. These factors substantially limit the feasibility of establishing community-based healthy pediatric MRI cohorts. For these reasons, the use of carefully selected clinical controls with structurally normal MRI findings is a widely accepted methodological approach in pediatric neuroimaging research [6,13,14]. This strategy has been frequently adopted in retrospective pediatric neuroimaging studies to address practical and ethical constraints associated with recruiting healthy pediatric participants [6,14]. Nevertheless, the possibility of residual confounding cannot be entirely excluded, and this limitation should be taken into account when considering the generalizability of the present findings to the broader pediatric population. Another methodological consideration relates to atlas suitability in pediatric neuroimaging. Although vol2Brain and FreeSurfer have been extensively utilized in pediatric neuroimaging research, the anatomical atlases and probabilistic models underlying these segmentation frameworks were primarily developed using adult or predominantly adult neuroanatomical datasets. Consequently, developmental differences between the study population and the atlas populations may influence segmentation performance and introduce subtle atlas-related bias. This issue is particularly relevant in automated morphometric analyses because neurodevelopmental processes continue throughout the pediatric developmental period, affecting cortical, subcortical, cerebellar, and thalamic structures. Therefore, the possibility of minor segmentation inaccuracies associated with atlas transferability should be acknowledged. However, the present study was restricted to individuals aged 12–17 years, a relatively advanced stage of neurodevelopment during which global brain morphology and the gross anatomical organization of major cerebral, cerebellar, and thalamic structures more closely resemble adult configurations than those observed in younger pediatric populations. This characteristic may reduce, although not completely eliminate, the potential impact of atlas-related anatomical mismatch. Furthermore, the segmentation frameworks used in the present study incorporate methodological features intended to improve anatomical adaptability. The FreeSurfer thalamic nuclei module employs a probabilistic atlas informed by high-resolution ex vivo MRI and histological delineation of thalamic nuclei, whereas vol2Brain utilizes multi-atlas, patch-based segmentation strategies combined with nonlinear registration procedures. These approaches are designed to accommodate interindividual anatomical variability and may reduce susceptibility to segmentation errors arising from anatomical differences between subjects and atlas templates. To further minimize the possibility of atlas-related inaccuracies, all segmentation outputs underwent systematic visual quality-control assessment using the native three-dimensional T1-weighted images. Segmentation masks were reviewed in axial, sagittal, and coronal planes to evaluate anatomical plausibility, boundary integrity, and consistency with individual anatomy. In addition, secondary quality-control assessment and interobserver reliability analysis demonstrated excellent reproducibility. No systematic atlas-related missegmentations were identified during these evaluations. Nevertheless, because a dedicated age-specific pediatric atlas was not used, subtle atlas-related segmentation inaccuracies cannot be entirely excluded. Accordingly, this issue should be considered when interpreting the morphometric findings and represents an inherent limitation of the present study. Another limitation relates to statistical power. Because this was a retrospective study, an a priori sample size calculation was not performed before participant inclusion. However, a post hoc power assessment based on the final study population of 74 patients with migraine without aura and 70 healthy controls demonstrated approximately 85% statistical power to detect a moderate effect size (d = 0.50) and approximately 90% power to detect an effect size of d = 0.54 at a two-sided significance level of α = 0.05. These findings suggest that the study was adequately powered to detect moderate morphometric differences between groups. Nevertheless, given the large number of investigated whole-brain, cerebellar, and thalamic measurements, smaller regional effects may not have been detected, particularly after correction for multiple comparisons. Therefore, although the present sample size was sufficient for detecting moderate group differences, the possibility of false-negative findings for subtle morphometric alterations cannot be entirely excluded. Future multicenter studies with larger cohorts may provide additional sensitivity for identifying smaller effect sizes and further validating the present findings. The use of a 1.5-T MRI system may also have limited the detection of subtle morphometric features that could potentially be identified using higher-field-strength imaging platforms. Finally, because the primary objective of the study was to evaluate structural morphometric differences between migraine and control groups, detailed analyses examining associations between imaging findings and clinical variables, such as disease duration, attack frequency, and other migraine characteristics, were not performed. Furthermore, standardized migraine severity and disability measures, such as the Pediatric Migraine Disability Assessment (PedMIDAS), Migraine Disability Assessment Scale (MIDAS), and Headache Impact Test (HIT-6), were not consistently available because of the retrospective study design, precluding more comprehensive clinical–imaging correlation analyses. Therefore, the present findings primarily reflect group-level neuroanatomical differences. Future multicenter longitudinal studies incorporating detailed clinical–imaging correlations will be necessary to clarify the relationship between morphometric alterations and clinical disease characteristics. Such investigations may contribute to a more comprehensive understanding of the neuroanatomical alterations and developmental morphometric changes associated with migraine without aura.

## 5. Conclusions

In this study, multiplatform morphometric neuroimaging analyses performed in adolescents aged 12–17 years with migraine without aura revealed morphometric alterations involving cortical, limbic, thalamic, and cerebellar structures. Among the most notable and distinctive findings were the volume reductions observed in the bilateral mediodorsal and pulvinar thalamic nuclei, bilateral Lobule VI, and the cortical thinning identified in the right Crus I and Crus II. The combined assessment of whole-brain morphometry, cortical thickness measurements, cerebellar subregional analyses, and thalamic nuclei segmentation suggests that migraine-related morphometric alterations in the developing brain are not confined to a single anatomical region but may involve a distributed pattern affecting multiple cortical, subcortical, and cerebellar structures. These findings support the concept that migraine may not be adequately explained by a localized neuroanatomical abnormality and may instead be associated with distributed morphometric differences involving cortical, limbic, thalamic, and cerebellar systems.

The focus on a neurodevelopmentally more homogeneous cohort of adolescents aged 12–17 years and the exclusive inclusion of patients with migraine without aura allowed the observed morphometric patterns to be evaluated within a more controlled developmental framework. Furthermore, the integrated use of vol2Brain, FreeSurfer, and 3D Slicer enabled a comprehensive multiplatform analysis encompassing multiple neuroanatomical systems. Nevertheless, the biological significance, clinical implications, and temporal evolution of the observed morphometric alterations cannot be determined from the current study design. Future longitudinal, multicenter, and multimodal neuroimaging investigations are therefore warranted to further elucidate the relationship between these structural alterations and migraine, as well as their potential neurodevelopmental significance.

In conclusion, the present findings contribute to the pediatric migraine neuroimaging literature and provide a foundation for future investigations focusing on the morphometric alterations observed in thalamic nuclei and cerebellar subregions. By integrating whole-brain morphometry, cerebellar subregional analysis, and thalamic nuclei segmentation within a neurodevelopmentally more homogeneous cohort of adolescents aged 12–17 years, our multiplatform approach enabled a more comprehensive characterization of migraine-associated neuroanatomical differences. Notably, the thalamic nuclei and cerebellar subregional findings that remained significant after correction for multiple comparisons suggest that these anatomical structures may represent particularly important targets for future validation studies.

## Figures and Tables

**Table 1:** Classification of thalamic nuclei included in the volumetric analyses according to the FreeSurfer Bayesian atlas-based thalamic nuclei segmentation framework. Major thalamic nuclear groups and their corresponding subnuclei are presented.

Group	Nuclei Included
Anterior	Anteroventral nucleus (AVN)
Intralaminar/Midline	Centromedian nucleus (CN), Midline thalamic nuclei (MTN), Intralaminar nuclei (ISN)
Medial	Mediodorsal nucleus (MN)
Posterior	Pulvinar nucleus (PN), Medial geniculate nucleus (MGN), Lateral geniculate nucleus (LGN)
Ventral	Ventral anterior nucleus (VAN), Ventrolateral anterior nucleus (VLAN), Ventrolateral posterior nucleus (VLPN), Ventral posterolateral nucleus (VPLN)
Habenular	Habenular nucleus (HN)
Whole Thalamus	Right and left thalamus

**Table 2:** Demographic characteristics and baseline clinical features of pediatric patients aged 12–17 years with migraine without aura and migraine-negative controls.

Variable	Migraine Without Aura (*n* = 74)	Control (*n* = 70)	*p*-Value
Age, years	14.8 ± 1.6	14.3 ± 1.5	0.071
Sex			0.337
Female, *n* (%)	46 (62.2)	38 (54.3)	
Male, *n* (%)	28 (37.8)	32 (45.7)	
Body mass index, kg/m^2^	21.4 ± 3.2	20.8 ± 3.0	0.249
Total intracranial volume, cm^3^	1398 ± 118	1432 ± 122	0.12
History of syncope/nonfocal complaint, *n* (%)	—	43 (61.4)	—
Disease duration, years	2.6 ± 1.4	—	—
Age at migraine onset, years	12.2 ± 1.8	—	—
Monthly attack frequency, attacks/month	4.1 ± 2.3	—	—
Attack duration, hours	13.6 ± 8.4	—	—
Nausea/vomiting, *n* (%)	42 (56.8)	—	—
Photophobia, *n* (%)	34 (45.9)	—	—
Phonophobia, *n* (%)	45 (60.8)	—	—
Unilateral headache, *n* (%)	49 (66.2)	—	—
Pulsating headache quality, *n* (%)	51 (68.9)	—	—
Analgesic use during attacks, *n* (%)	58 (78.4)	—	—

Values are presented as mean ± standard deviation or *n* (%), as appropriate. Total intracranial volume (TIV) is reported in cm^3^ and represents the intracranial cavity volume derived from the Vol2Brain output. *p*-value represents comparisons between the migraine and control groups. Variables specific to migraine were not compared with controls. The symbol ‘—’ indicates that no patients in the corresponding group had the relevant clinical characteristic.

**Table 3:** Group comparisons of significant morphometric parameters derived from whole-brain automated MRI analysis after false discovery rate (FDR) correction.

Anatomical Region	Measurement Type	Migraine Without Aura Mean ± SD	Control Mean ± SD	*p*-Value	q (FDR)	Effect Size (Hedges’ g) [95% CI]
Amygdala (R)	Volume (mm^3^)	1712 ± 221	1846 ± 238	0.004	0.029	0.58 [0.25, 0.92]
Amygdala (L)	Volume (mm^3^)	1695 ± 214	1818 ± 226	0.006	0.032	0.56 [0.22, 0.89]
Anterior Insula (R)	Cortical Thickness (mm)	3.37 ± 0.24	3.52 ± 0.25	0.009	0.038	0.61 [0.27, 0.95]
Anterior Cingulate Gyrus (L)	Cortical Thickness (mm)	4.01 ± 0.31	4.19 ± 0.30	0.012	0.041	0.59 [0.25, 0.93]
Precuneus	Cortical Thickness (mm)	2.81 ± 0.27	2.96 ± 0.28	0.016	0.045	0.54 [0.21, 0.88]
Thalamus (R)	Volume (mm^3^)	5635 ± 612	5878 ± 641	0.026	0.049	0.39 [0.06, 0.72]
Remaining 129 regions	—	—	—	N.S.	—	—

Values are presented as mean ± standard deviation (SD). *p*-value represents between-group comparisons for each anatomical region. Multiple-comparison correction was performed using the Benjamini–Hochberg false discovery rate (FDR) procedure, and regions with q < 0.05 were considered statistically significant. Effect sizes are reported as Hedges’ g with 95% confidence intervals (CI). Positive Hedges’ g values indicate lower measurements in the migraine group relative to controls. Only regions remaining significant after FDR correction are shown. Abbreviations: SD, standard deviation; CI, confidence interval; FDR, false discovery rate. “N.S.”, not significant; “—”, results not individually reported because no statistically significant difference was detected.

**Table 4:** Group comparisons of cerebellar subregional volumes obtained from automated cerebellar segmentation after false discovery rate (FDR) correction.

Anatomical Region	Side	Migraine Without Aura Mean ± SD (cm^3^)	Control Mean ± SD (cm^3^)	*p*-Value	q (FDR)	Effect Size Hedges’ g [95% CI]
Lobule I–II	Right	0.060 ± 0.009	0.061 ± 0.008	0.72	0.86	0.05 [−0.34, 0.44]
Lobule I–II	Left	0.051 ± 0.008	0.053 ± 0.008	0.76	0.88	0.04 [−0.35, 0.43]
Lobule III	Right	0.61 ± 0.08	0.63 ± 0.08	0.49	0.72	0.11 [−0.28, 0.50]
Lobule III	Left	0.56 ± 0.07	0.58 ± 0.08	0.53	0.75	0.10 [−0.29, 0.49]
Lobule IV	Right	1.84 ± 0.19	1.89 ± 0.20	0.41	0.68	0.14 [−0.25, 0.53]
Lobule IV	Left	2.18 ± 0.22	2.24 ± 0.23	0.38	0.65	0.15 [−0.24, 0.54]
Lobule V	Right	3.94 ± 0.37	4.03 ± 0.39	0.36	0.63	0.17 [−0.22, 0.56]
Lobule V	Left	4.01 ± 0.38	4.08 ± 0.40	0.40	0.66	0.16 [−0.23, 0.55]
Lobule VI	**Right**	**8.88 ± 0.71**	**9.29 ± 0.76**	**0.011**	**0.041**	**0.46 [0.12, 0.80]**
Lobule VI	**Left**	**8.93 ± 0.69**	**9.35 ± 0.74**	**0.014**	**0.046**	**0.43 [0.09, 0.77]**
Lobule VIIA (Crus I)	Right	11.24 ± 1.08	11.46 ± 1.12	0.18	0.43	0.20 [−0.19, 0.59]
Lobule VIIA (Crus I)	Left	11.31 ± 1.10	11.55 ± 1.14	0.21	0.48	0.19 [−0.20, 0.58]
Lobule VIIA (Crus II)	Right	7.08 ± 0.74	7.23 ± 0.76	0.23	0.50	0.18 [−0.21, 0.57]
Lobule VIIA (Crus II)	Left	7.14 ± 0.75	7.28 ± 0.77	0.28	0.57	0.16 [−0.23, 0.55]
Lobule VIIB	Right	4.71 ± 0.46	4.82 ± 0.47	0.31	0.59	0.16 [−0.23, 0.55]
Lobule VIIB	Left	5.02 ± 0.49	5.12 ± 0.50	0.34	0.61	0.15 [−0.24, 0.54]
Lobule VIIIA	Right	6.32 ± 0.61	6.45 ± 0.63	0.35	0.63	0.14 [−0.25, 0.53]
Lobule VIIIA	Left	5.71 ± 0.56	5.83 ± 0.58	0.37	0.65	0.13 [−0.26, 0.52]
Lobule VIIIB	Right	3.98 ± 0.39	4.07 ± 0.40	0.054	0.081	0.30 [−0.09, 0.69]
Lobule VIIIB	Left	3.58 ± 0.35	3.66 ± 0.36	0.40	0.66	0.14 [−0.25, 0.53]
Lobule IX	Right	4.08 ± 0.42	4.16 ± 0.43	0.51	0.73	0.10 [−0.29, 0.49]
Lobule IX	Left	3.80 ± 0.39	3.88 ± 0.40	0.55	0.76	0.09 [−0.30, 0.48]
Lobule X	Right	0.62 ± 0.08	0.64 ± 0.08	0.45	0.69	0.12 [−0.27, 0.51]
Lobule X	Left	0.59 ± 0.07	0.61 ± 0.08	0.48	0.71	0.11 [−0.28, 0.50]

Values are presented as mean ± standard deviation (SD). *p*-value represents between-group comparisons for each cerebellar subregion. Multiple-comparison correction was performed using the Benjamini–Hochberg false discovery rate (FDR) procedure, and regions with q < 0.05 were considered statistically significant. Effect sizes are reported as Hedges’ g with 95% confidence intervals (CI). Positive Hedges’ g values indicate lower measurements in the migraine group relative to controls. Statistically significant findings are shown in bold. Abbreviations: SD, standard deviation; CI, confidence interval; FDR, false discovery rate.

**Table 5:** Group comparisons of cerebellar subregional gray matter volumes obtained from automated cerebellar segmentation after false discovery rate (FDR) correction.

Anatomical Region	Side	Migraine Without Aura Mean ± SD (cm^3^)	Control Mean ± SD (cm^3^)	*p*-Value	q (FDR)	Effect Size Hedges’ g [95% CI]
Lobule I–II	Right	0.035 ± 0.006	0.036 ± 0.006	0.68	0.84	0.07 [−0.26, 0.40]
Lobule I–II	Left	0.022 ± 0.005	0.023 ± 0.005	0.72	0.86	0.05 [−0.28, 0.38]
Lobule III	Right	0.53 ± 0.07	0.55 ± 0.07	0.44	0.68	0.13 [−0.20, 0.46]
Lobule III	Left	0.49 ± 0.07	0.51 ± 0.07	0.47	0.70	0.12 [−0.21, 0.45]
Lobule IV	Right	1.69 ± 0.18	1.75 ± 0.19	0.28	0.55	0.19 [−0.14, 0.52]
Lobule IV	Left	2.03 ± 0.21	2.10 ± 0.22	0.25	0.52	0.20 [−0.13, 0.53]
Lobule V	Right	3.41 ± 0.33	3.52 ± 0.35	0.22	0.48	0.22 [−0.11, 0.55]
Lobule V	Left	3.55 ± 0.34	3.66 ± 0.36	0.24	0.50	0.21 [−0.12, 0.54]
Lobule VI	**Right**	**8.19 ± 0.64**	**8.73 ± 0.68**	**0.003**	**0.018**	**0.64 [0.30, 0.98]**
Lobule VI	**Left**	**8.24 ± 0.63**	**8.78 ± 0.67**	**0.005**	**0.023**	**0.60 [0.26, 0.94]**
Lobule VIIA (Crus I)	Right	9.86 ± 0.91	10.01 ± 0.94	0.19	0.45	0.23 [−0.10, 0.56]
Lobule VIIA (Crus I)	Left	9.95 ± 0.92	10.10 ± 0.95	0.21	0.47	0.22 [−0.11, 0.55]
Lobule VIIA (Crus II)	Right	6.16 ± 0.66	6.28 ± 0.68	0.16	0.42	0.24 [−0.09, 0.57]
Lobule VIIA (Crus II)	Left	6.55 ± 0.69	6.67 ± 0.70	0.18	0.44	0.23 [−0.10, 0.56]
Lobule VIIB	Right	4.48 ± 0.44	4.58 ± 0.45	0.26	0.53	0.19 [−0.14, 0.52]
Lobule VIIB	Left	4.55 ± 0.44	4.64 ± 0.46	0.31	0.58	0.17 [−0.16, 0.50]
Lobule VIIIA	Right	5.84 ± 0.56	5.96 ± 0.58	0.24	0.51	0.20 [−0.13, 0.53]
Lobule VIIIA	Left	5.47 ± 0.53	5.58 ± 0.55	0.29	0.56	0.18 [−0.15, 0.51]
Lobule VIIIB	Right	3.54 ± 0.35	3.63 ± 0.36	0.061	0.088	0.29 [−0.04, 0.62]
Lobule VIIIB	Left	3.24 ± 0.33	3.31 ± 0.34	0.34	0.60	0.16 [−0.17, 0.49]
Lobule IX	Right	3.56 ± 0.37	3.65 ± 0.38	0.39	0.64	0.14 [−0.19, 0.47]
Lobule IX	Left	3.25 ± 0.34	3.33 ± 0.35	0.42	0.66	0.13 [−0.20, 0.46]
Lobule X	Right	0.60 ± 0.08	0.62 ± 0.08	0.33	0.59	0.16 [−0.17, 0.49]
Lobule X	Left	0.56 ± 0.07	0.58 ± 0.07	0.37	0.62	0.15 [−0.18, 0.48]

Values are presented as mean ± standard deviation (SD). *p*-value represents between-group comparisons of cerebellar subregional gray matter volumes. Multiple-comparison correction was performed using the Benjamini–Hochberg false discovery rate (FDR) procedure, and regions with q < 0.05 were considered statistically significant. Effect sizes are reported as Hedges’ g with corresponding 95% confidence intervals (CI). Positive Hedges’ g values indicate lower measurements in the migraine group relative to controls. Statistically significant findings are highlighted in bold. Abbreviations: SD, standard deviation; CI, confidence interval; FDR, false discovery rate.

**Table 6:** Group comparisons of cerebellar cortical thickness measurements obtained from automated cerebellar segmentation after false discovery rate (FDR) correction.

Anatomical Region	Side	Migraine Without Aura Mean ± SD (mm)	Control Mean ± SD (mm)	*p*-Value	q (FDR)	Effect Size Hedges’ g [95% CI]
Lobule I–II	Right	3.22 ± 0.36	3.26 ± 0.37	0.54	0.72	0.10 [−0.23, 0.43]
Lobule I–II	Left	3.14 ± 0.35	3.18 ± 0.36	0.51	0.70	0.11 [−0.22, 0.44]
Lobule III	Right	3.91 ± 0.40	3.97 ± 0.41	0.39	0.61	0.15 [−0.18, 0.48]
Lobule III	Left	4.12 ± 0.41	4.18 ± 0.42	0.42	0.64	0.14 [−0.19, 0.47]
Lobule IV	Right	5.21 ± 0.43	5.28 ± 0.45	0.33	0.56	0.16 [−0.17, 0.49]
Lobule IV	Left	5.19 ± 0.44	5.26 ± 0.45	0.35	0.58	0.15 [−0.18, 0.48]
Lobule V	Right	4.72 ± 0.38	4.80 ± 0.39	0.26	0.49	0.20 [−0.13, 0.53]
Lobule V	Left	4.87 ± 0.40	4.94 ± 0.41	0.32	0.55	0.17 [−0.16, 0.50]
Lobule VI	Right	4.96 ± 0.36	5.05 ± 0.37	0.17	0.38	0.24 [−0.09, 0.57]
Lobule VI	Left	5.01 ± 0.37	5.09 ± 0.38	0.21	0.44	0.21 [−0.12, 0.54]
Lobule VIIA (Crus I)	**Right**	**4.84 ± 0.31**	**5.06 ± 0.34**	**0.006**	**0.031**	**0.66 [0.32, 1.00]**
Lobule VIIA (Crus I)	Left	4.91 ± 0.33	4.99 ± 0.34	0.18	0.39	0.23 [−0.10, 0.56]
Lobule VIIA (Crus II)	**Right**	**4.72 ± 0.30**	**4.91 ± 0.32**	**0.011**	**0.044**	**0.61 [0.27, 0.95]**
Lobule VIIA (Crus II)	Left	4.78 ± 0.31	4.85 ± 0.33	0.25	0.48	0.21 [−0.12, 0.54]
Lobule VIIB	Right	5.05 ± 0.37	5.13 ± 0.38	0.20	0.43	0.21 [−0.12, 0.54]
Lobule VIIB	Left	5.03 ± 0.36	5.10 ± 0.37	0.28	0.52	0.19 [−0.14, 0.52]
Lobule VIIIA	Right	4.91 ± 0.35	4.98 ± 0.36	0.29	0.53	0.18 [−0.15, 0.51]
Lobule VIIIA	Left	5.03 ± 0.36	5.10 ± 0.37	0.30	0.54	0.18 [−0.15, 0.51]
Lobule VIIIB	Right	4.93 ± 0.34	5.01 ± 0.36	0.19	0.41	0.22 [−0.11, 0.55]
Lobule VIIIB	Left	5.09 ± 0.36	5.15 ± 0.37	0.36	0.59	0.16 [−0.17, 0.49]
Lobule IX	Right	4.96 ± 0.39	5.02 ± 0.40	0.43	0.65	0.14 [−0.19, 0.47]
Lobule IX	Left	4.99 ± 0.40	5.04 ± 0.41	0.48	0.68	0.12 [−0.21, 0.45]
Lobule X	Right	3.91 ± 0.37	3.98 ± 0.38	0.34	0.57	0.17 [−0.16, 0.50]
Lobule X	Left	3.96 ± 0.38	4.02 ± 0.39	0.41	0.63	0.15 [−0.18, 0.48]

Values are presented as mean ± standard deviation (SD). *p*-value represents between-group comparisons for each cerebellar cortical thickness measurement. Multiple-comparison correction was performed using the Benjamini–Hochberg false discovery rate (FDR) procedure, and regions with q < 0.05 were considered statistically significant. Effect sizes are reported as Hedges’ g with 95% confidence intervals (CI). Positive Hedges’ g values indicate lower measurements in the migraine group relative to controls. Statistically significant findings are shown in bold. Abbreviations: SD, standard deviation; CI, confidence interval; FDR, false discovery rate.

**Table 7:** Group comparisons of thalamic nuclei volumes obtained from automated thalamic nuclei segmentation after false discovery rate (FDR) correction.

Anatomical Region	Side	Migraine Without Aura Mean ± SD (cm^3^)	Control Mean ± SD (cm^3^)	*p*-Value	q (FDR)	Effect Size Hedges’ g [95% CI]
AVN	Right	0.101 ± 0.018	0.105 ± 0.019	0.28	0.56	0.18 [−0.15, 0.51]
AVN	Left	0.099 ± 0.017	0.103 ± 0.018	0.34	0.62	0.15 [−0.18, 0.48]
VAN	**Right**	**0.232 ± 0.036**	**0.252 ± 0.039**	**0.014**	**0.041**	**0.53 [0.19, 0.87]**
VAN	Left	0.229 ± 0.035	0.242 ± 0.038	0.071	0.108	0.31 [−0.02, 0.64]
VLAN	Right	0.091 ± 0.014	0.095 ± 0.015	0.22	0.49	0.22 [−0.11, 0.55]
VLAN	Left	0.096 ± 0.014	0.101 ± 0.015	0.18	0.44	0.25 [−0.08, 0.58]
VLPN	Right	0.795 ± 0.078	0.820 ± 0.083	0.083	0.127	0.29 [−0.04, 0.62]
VLPN	Left	0.808 ± 0.079	0.834 ± 0.084	0.092	0.136	0.28 [−0.05, 0.61]
VPLN	**Right**	**0.281 ± 0.041**	**0.305 ± 0.045**	**0.011**	**0.038**	**0.56 [0.22, 0.90]**
VPLN	Left	0.314 ± 0.044	0.329 ± 0.047	0.081	0.122	0.29 [−0.04, 0.62]
PN	**Right**	**1.108 ± 0.126**	**1.196 ± 0.132**	**0.006**	**0.027**	**0.68 [0.34, 1.02]**
PN	**Left**	**1.236 ± 0.135**	**1.321 ± 0.142**	**0.009**	**0.034**	**0.61 [0.27, 0.95]**
LGN	Right	0.097 ± 0.018	0.101 ± 0.019	0.31	0.60	0.16 [−0.17, 0.49]
LGN	Left	0.088 ± 0.016	0.091 ± 0.017	0.39	0.66	0.13 [−0.20, 0.46]
MGN	Right	0.076 ± 0.011	0.079 ± 0.012	0.27	0.55	0.18 [−0.15, 0.51]
MGN	Left	0.067 ± 0.010	0.070 ± 0.011	0.25	0.53	0.19 [−0.14, 0.52]
CN	Right	0.118 ± 0.017	0.123 ± 0.018	0.19	0.45	0.24 [−0.09, 0.57]
CN	Left	0.116 ± 0.017	0.121 ± 0.018	0.23	0.50	0.22 [−0.11, 0.55]
MN	**Right**	**0.615 ± 0.071**	**0.664 ± 0.076**	**0.004**	**0.021**	**0.66 [0.32, 1.00]**
MN	**Left**	**0.618 ± 0.070**	**0.660 ± 0.074**	**0.012**	**0.039**	**0.58 [0.24, 0.92]**
HN	Right	0.019 ± 0.004	0.020 ± 0.004	0.42	0.68	0.12 [−0.21, 0.45]
HN	Left	0.020 ± 0.004	0.021 ± 0.004	0.45	0.70	0.11 [−0.22, 0.44]
MTN	Right	0.010 ± 0.003	0.011 ± 0.003	0.48	0.72	0.10 [−0.23, 0.43]
MTN	Left	0.011 ± 0.003	0.012 ± 0.003	0.51	0.74	0.09 [−0.24, 0.42]
ISN	Right	2.170 ± 0.210	2.218 ± 0.224	0.19	0.45	0.23 [−0.10, 0.56]
ISN	Left	2.158 ± 0.208	2.205 ± 0.219	0.21	0.48	0.22 [−0.11, 0.55]

Values are presented as mean ± standard deviation (SD). *p*-value represents between-group comparisons for each thalamic nucleus. Multiple-comparison correction was performed using the Benjamini–Hochberg false discovery rate (FDR) procedure, and nuclei with q < 0.05 were considered statistically significant. Effect sizes are reported as Hedges’ g with 95% confidence intervals (CI). Positive Hedges’ g values indicate lower measurements in the migraine group relative to controls. Statistically significant findings are shown in bold. Abbreviations: SD, standard deviation; CI, confidence interval; FDR, false discovery rate; AVN, anteroventral nucleus; VAN, ventral anterior nucleus; VLAN, ventrolateral anterior nucleus; VLPN, ventrolateral posterior nucleus; VPLN, ventral posterolateral nucleus; PN, pulvinar nucleus; LGN, lateral geniculate nucleus; MGN, medial geniculate nucleus; CN, centromedian nucleus; MN, mediodorsal nucleus; HN, habenular nucleus; MTN, midline thalamic nuclei; ISN, intralaminar nuclei.

**Table 8:** TIV-adjusted volumetric findings that remained significant after false discovery rate (FDR) correction.

Anatomical Region	Measurement Type	Migraine Without Aura Mean ± SD	Control Mean ± SD	*p*-Value	q (FDR)	Effect Size Hedges’ g [95% CI]
Right Amygdala	TIV-adjusted volume (cm^3^)	1.70 ± 0.21	1.83 ± 0.24	0.011	0.032	0.55 [0.22, 0.88]
Left Amygdala	TIV-adjusted volume (cm^3^)	1.68 ± 0.20	1.80 ± 0.22	0.014	0.037	0.52 [0.19, 0.85]
Right Thalamus	TIV-adjusted volume (cm^3^)	5.60 ± 0.60	5.84 ± 0.64	0.021	0.046	0.42 [0.09, 0.75]
Right Lobule VI	TIV-adjusted volume (cm^3^)	8.84 ± 0.70	9.25 ± 0.75	0.017	0.041	0.45 [0.12, 0.78]
Left Lobule VI	TIV-adjusted volume (cm^3^)	8.89 ± 0.69	9.30 ± 0.74	0.019	0.044	0.43 [0.10, 0.76]
Right Lobule VI	TIV-adjusted gray matter volume (cm^3^)	8.15 ± 0.63	8.68 ± 0.67	0.005	0.019	0.62 [0.29, 0.95]
Left Lobule VI	TIV-adjusted gray matter volume (cm^3^)	8.20 ± 0.62	8.73 ± 0.66	0.007	0.024	0.59 [0.26, 0.92]
Right VAN	TIV-adjusted volume (cm^3^)	0.229 ± 0.035	0.249 ± 0.038	0.018	0.043	0.50 [0.17, 0.83]
Right VPLN	TIV-adjusted volume (cm^3^)	0.278 ± 0.040	0.302 ± 0.044	0.014	0.036	0.54 [0.21, 0.87]
Right PN	TIV-adjusted volume (cm^3^)	1.097 ± 0.123	1.184 ± 0.129	0.008	0.026	0.66 [0.33, 0.99]
Left PN	TIV-adjusted volume (cm^3^)	1.225 ± 0.132	1.309 ± 0.139	0.011	0.031	0.60 [0.27, 0.93]
Right MN	TIV-adjusted volume (cm^3^)	0.608 ± 0.070	0.656 ± 0.074	0.006	0.021	0.64 [0.31, 0.97]
Left MN	TIV-adjusted volume (cm^3^)	0.611 ± 0.069	0.652 ± 0.073	0.013	0.034	0.57 [0.24, 0.90]

Values are presented as mean ± standard deviation (SD). Volumetric parameters were adjusted for total intracranial volume (TIV) using the following formula: TIV-adjusted volume = regional volume/individual TIV × mean cohort TIV. *p*-value represents between-group comparisons of TIV-adjusted volumetric measures. Multiple-comparison correction was performed using the Benjamini–Hochberg false discovery rate (FDR) procedure, and regions with q < 0.05 were considered statistically significant. Effect sizes are reported as Hedges’ g with 95% confidence intervals (CI). Positive Hedges’ g values indicate lower measurements in the migraine group relative to controls. Only volumetric parameters that remained significant after TIV adjustment and FDR correction are shown. Cortical thickness parameters were not subjected to TIV adjustment because they are non-volumetric measures and are not directly influenced by intracranial volume. Abbreviations: SD, standard deviation; CI, confidence interval; TIV, total intracranial volume; FDR, false discovery rate; VAN, ventral anterior nucleus; VPLN, ventral posterolateral nucleus; PN, pulvinar nucleus; MN, mediodorsal nucleus.

**Table 9:** Summary of morphometric findings remaining statistically significant after Benjamini–Hochberg false discovery rate correction.

Anatomical Domain	Anatomical Region	Measurement Type	Direction of Change in Migraine Group	q (FDR)	Effect Size (Hedges’ g) [95% CI]
Whole-brain morphometry	Right amygdala	Volume	↓	0.029	0.58 [0.25, 0.92]
Whole-brain morphometry	Left amygdala	Volume	↓	0.032	0.56 [0.22, 0.89]
Whole-brain morphometry	Right thalamus	Volume	↓	0.049	0.39 [0.06, 0.72]
Whole-brain morphometry	Right anterior insula	Cortical thickness	↓	0.038	0.61 [0.27, 0.95]
Whole-brain morphometry	Left anterior cingulate gyrus	Cortical thickness	↓	0.041	0.59 [0.25, 0.93]
Whole-brain morphometry	Precuneus	Cortical thickness	↓	0.045	0.54 [0.21, 0.88]
Cerebellar subregional analysis	Right Lobule VI	Volume	↓	0.041	0.46 [0.12, 0.80]
Cerebellar subregional analysis	Left Lobule VI	Volume	↓	0.046	0.43 [0.09, 0.77]
Cerebellar subregional analysis	Right Lobule VI	Gray matter volume	↓	0.018	0.64 [0.30, 0.98]
Cerebellar subregional analysis	Left Lobule VI	Gray matter volume	↓	0.023	0.60 [0.26, 0.94]
Cerebellar subregional analysis	Right Crus I	Cortical thickness	↓	0.031	0.66 [0.32, 1.00]
Cerebellar subregional analysis	Right Crus II	Cortical thickness	↓	0.044	0.61 [0.27, 0.95]
Thalamic nuclei analysis	Right ventral anterior nucleus (VAN)	Volume	↓	0.041	0.53 [0.19, 0.87]
Thalamic nuclei analysis	Right ventral posterolateral nucleus (VPLN)	Volume	↓	0.038	0.56 [0.22, 0.90]
Thalamic nuclei analysis	Right pulvinar nucleus (PN)	Volume	↓	0.027	0.68 [0.34, 1.02]
Thalamic nuclei analysis	Left pulvinar nucleus (PN)	Volume	↓	0.034	0.61 [0.27, 0.95]
Thalamic nuclei analysis	Right mediodorsal nucleus (MN)	Volume	↓	0.021	0.66 [0.32, 1.00]
Thalamic nuclei analysis	Left mediodorsal nucleus (MN)	Volume	↓	0.039	0.58 [0.24, 0.92]

Values shown represent only findings that remained statistically significant after Benjamini–Hochberg false discovery rate (FDR) correction. Direction of change refers to lower measurements in the migraine group relative to controls. Effect sizes are reported as Hedges’ g. Positive Hedges’ g values indicate lower morphometric measurements in patients with migraine without aura compared with migraine-negative controls. q values represent FDR-adjusted significance levels. Abbreviations: FDR, false discovery rate; VAN, ventral anterior nucleus; VPLN, ventral posterolateral nucleus; PN, pulvinar nucleus; MN, mediodorsal nucleus. “↓” indicates reduced measurements in the migraine group relative to the control group.

## Data Availability

The data presented in this study are available on request from the corresponding author due to ethical and privacy restrictions.

## Supplementary Materials

The following supporting information can be downloaded at: https://www.mdpi.com/article/10.3390/diagnostics16132085/s1. Supplementary Material S1. Representative output of automated global brain volumetric analysis generated using the Vol2Brain pipeline from a high-resolution isotropic 3D T1-weighted MRI examination of a pediatric patient with migraine without aura. Supplementary Material S2. Representative output of automated cerebellar subregional volumetric analysis generated using the Vol2Brain pipeline from a high-resolution isotropic 3D T1-weighted MRI examination of a pediatric patient with migraine without aura. Supplementary Material S3. Computational pipeline for FreeSurfer-based thalamic nuclei segmentation. Supplementary Material S4. Confirmatory ANCOVA analyses of morphometric findings that remained statistically significant after primary FDR correction.

## References

[1] Khan A., Liu S., Tao F. (2025). Current trends in pediatric migraine: Clinical insights and therapeutic strategies. Brain Sci..

[2] Onofri A., Pensato U., Rosignoli C., Caponnetto V., Charlotte C., Gabriel M., Ornello R., Sacco S. (2023). Primary headache epidemiology in children and adolescents: A systematic review and meta-analysis. J. Headache Pain.

[3] Konrad K., Firk C., Uhlhaas P.J. (2013). Brain development during adolescence: Neuroscientific insights into this developmental period. Dtsch. Arztebl. Int..

[4] Rocca M.A., Messina R., Colombo B., Falini A., Comi G., Filippi M. (2014). Structural brain MRI abnormalities in pediatric patients with migraine. J. Neurol..

[5] Xu W.J., Barisano G., Phung D., Chou B., Pinto S.N., Lerner A., Sheikh-Bahaei N. (2023). Structural MRI in migraine: A review of migraine vascular and structural changes in brain parenchyma. J. Cent. Nerv. Syst. Dis..

[6] Bell T., Khaira A., Stokoe M., Webb M., Noel M., Amoozegar F., Harris A.D. (2021). Age-related differences in resting state functional connectivity in pediatric migraine. J. Headache Pain.

[7] Goadsby P.J., Holland P.R., Martins-Oliveira M., Hoffmann J., Schankin C., Akerman S. (2017). Pathophysiology of migraine: A disorder of sensory processing. Physiol. Rev..

[8] Zhou Y., Gong L., Yang Y., Zhang X., Liu J., Wang C., Zhang J., Wei X., Yu S. (2023). Spatio-temporal dynamics of resting-state brain networks are associated with migraine disability. J. Headache Pain.

[9] Schramm S., Börner C., Reichert M., Baum T., Zimmer C., Heinen F., Bonfert M.V., Sollmann N. (2023). Functional magnetic resonance imaging in migraine: A systematic review. Cephalalgia.

[10] Deodato M., Granato A., Martini M., Sabot R., Buoite Stella A., Manganotti P. (2024). Instrumental assessment of pressure pain threshold over trigeminal and extra-trigeminal area in people with episodic and chronic migraine: A cross-sectional observational study. Neurol. Sci..

[11] Coppola G., Di Lorenzo C., Schoenen J., Pierelli F. (2013). Habituation and sensitization in primary headaches. J. Headache Pain.

[12] Noseda R. (2022). Cerebro-cerebellar networks in migraine symptoms and headache. Front. Pain Res..

[13] Guarnera A., Bottino F., Napolitano A., Sforza G., Cappa M., Chioma L., Pasquini L., Rossi-Espagnet M.C., Lucignani G., Figà-Talamanca L. (2021). Early alterations of cortical thickness and gyrification in migraine without aura: A retrospective MRI study in pediatric patients. J. Headache Pain.

[14] Webb M.E., Amoozegar F., Harris A.D. (2019). Magnetic resonance imaging in pediatric migraine. Can. J. Neurol. Sci..

[15] Rudolph S., Badura A., Lutzu S., Pathak S.S., Thieme A., Verpeut J.L., Wagner M.J., Yang Y.M., Fioravante D. (2023). Cognitive-affective functions of the cerebellum. J. Neurosci..

[16] Schwedt T.J., Chong C.D., Chiang C.C., Baxter L., Schlaggar B.L., Dodick D.W. (2014). Enhanced pain-induced activity of pain-processing regions in a case-control study of episodic migraine. Cephalalgia.

[17] Guell X., Schmahmann J. (2020). Cerebellar functional anatomy: A didactic summary based on human fMRI evidence. Cerebellum.

[18] Akerman S., Holland P.R., Goadsby P.J. (2011). Diencephalic and brainstem mechanisms in migraine. Nat. Rev. Neurosci..

[19] Akçay H.İ. (2026). Comparison of brain volumes in episodic and chronic migraine using automated whole-brain volumetry. Front. Neurol..

[20] Harkey T., Baker D., Hagen J., Scott H., Palys V. (2022). Practical methods for segmentation and calculation of brain volume and intracranial volume: A guide and comparison. Quant. Imaging Med. Surg..

[21] Lee J., Lee J.Y., Oh S.W., Chung M.S., Park J.E., Moon Y., Jeon H.J., Moon W.J. (2021). Evaluation of reproducibility of brain volumetry between commercial software, Inbrain and established research purpose method, FreeSurfer. J. Clin. Neurol..

[22] Monereo-Sánchez J., de Jong J.J.A., Drenthen G.S., Beran M., Backes W.H., Stehouwer C.D.A., Schram M.T., Linden D.E.J., Jansen J.F.A. (2021). Quality control strategies for brain MRI segmentation and parcellation: Practical approaches and recommendations—Insights from the Maastricht study. NeuroImage.

[23] Herting M.M., Sowell E.R. (2017). Puberty and structural brain development in humans. Front. Neuroendocrinol..

[24] Arain M., Haque M., Johal L., Mathur P., Nel W., Rais A., Sandhu R., Sharma S. (2013). Maturation of the adolescent brain. Neuropsychiatr. Dis. Treat..

[25] Blakemore S.J. (2012). Imaging brain development: The adolescent brain. NeuroImage.

[26] Asato M.R., Terwilliger R., Woo J., Luna B. (2010). White matter development in adolescence: A DTI study. Cereb. Cortex.

[27] Headache Classification Committee of the International Headache Society (2018). The International Classification of Headache Disorders, 3rd edition. Cephalalgia.

[28] Mills K.L., Goddings A.L., Herting M.M., Meuwese R., Blakemore S.J., Crone E.A., Dahl R.E., Güroğlu B., Raznahan A., Sowell E.R. (2016). Structural brain development between childhood and adulthood: Convergence across four longitudinal samples. NeuroImage.

[29] Evans A.C., Brain Development Cooperative Group (2006). The NIH MRI study of normal brain development. NeuroImage.

[30] Giedd J.N., Blumenthal J., Jeffries N.O., Castellanos F.X., Liu H., Zijdenbos A., Paus T., Evans A.C., Rapoport J.L. (1999). Brain development during childhood and adolescence: A longitudinal MRI study. Nat. Neurosci..

[31] Šišić N., Rogelj P. (2025). Deep learning for brain MRI tissue and structure segmentation: A comprehensive review. Algorithms.

[32] Manjón J.V., Coupé P. (2016). volBrain: An online MRI brain volumetry system. Front. Neuroinform..

[33] Iglesias J.E., Insausti R., Lerma-Usabiaga G., Bocchetta M., Van Leemput K., Greve D.N., van der Kouwe A., Alzheimer’s Disease Neuroimaging Initiative, Fischl B., Caballero-Gaudes C. (2018). A probabilistic atlas of the human thalamic nuclei combining ex vivo MRI and histology. NeuroImage.

[34] Gaser C., Dahnke R., Thompson P.M., Kurth F., Luders E. (2024). CAT: A computational anatomy toolbox for the analysis of structural MRI data. GigaScience.

[35] Bürkle E., Nazzal A., Debolski A., Ernemann U., Lindig T., Bender B. (2025). Scan–rescan reliability assessment of brain volumetric analysis across scanners and software solutions. Sci. Rep..

[36] Li X., Morgan P.S., Ashburner J., Smith J., Rorden C. (2016). The first step for neuroimaging data analysis: DICOM to NIfTI conversion. J. Neurosci. Methods.

[37] Rushmore R.J., Bouix S., Kubicki M., Rathi Y., Yeterian E., Makris N. (2022). HOA2.0-ComPaRe: A next generation Harvard–Oxford Atlas comparative parcellation reasoning method for human and macaque individual brain parcellation and atlases of the cerebral cortex. Front. Neuroanat..

[38] Çetin S. (2024). Evaluation of brain structures’ volume using vol2Brain software in patients with idiopathic sudden sensorineural hearing loss. Indian J. Otol..

[39] Fischl B. (2012). FreeSurfer. NeuroImage.

[40] Dale A.M., Fischl B., Sereno M.I. (1999). Cortical surface-based analysis: I. Segmentation and surface reconstruction. NeuroImage.

[41] Fedorov A., Beichel R., Kalpathy-Cramer J., Finet J., Fillion-Robin J.C., Pujol S., Bauer C., Jennings D., Fennessy F., Sonka M. (2012). 3D Slicer as an image computing platform for the Quantitative Imaging Network. Magn. Reson. Imaging.

[42] de Tommaso M., Vecchio E., Quitadamo S.G., Coppola G., Di Renzo A., Parisi V., Silvestro M., Russo A., Tedeschi G. (2021). Pain-related brain connectivity changes in migraine: A narrative review and proof of concept about possible novel treatments interference. Brain Sci..

[43] Karsan N., Goadsby P.J. (2023). Neuroimaging in the pre-ictal or premonitory phase of migraine: A narrative review. J. Headache Pain.

[44] Matoso A., Fouto A.R., Esteves I., Caetano G., Vilela P., Gil-Gouveia R., Figueiredo P. (2024). Involvement of the cerebellum in structural connectivity enhancement in episodic migraine. J. Headache Pain.

[45] Casillo F., Sebastianelli G., Abagnale C., Di Renzo A., Ziccardi L., Parisi V., Coppola G. (2025). Brain imaging in migraine with and without aura: Similarities and differences. Cephalalgia.

[46] Bethlehem R.A.I., Seidlitz J., White S.R., Vogel J.W., Anderson K.M., Adamson C., Adler S., Alexopoulos G.S., Anagnostou E., Areces-Gonzalez A. (2022). Brain charts for the human lifespan. Nature.

[47] Buyanova I.S., Arsalidou M. (2021). Cerebral white matter myelination and relations to age, gender, and cognition: A selective review. Front. Hum. Neurosci..

[48] Liang X., Sun L., Liao X., Lei T., Xia M., Duan D., Zeng Z., Li Q., Xu Z., Men W. (2024). Structural connectome architecture shapes the maturation of cortical morphology from childhood to adolescence. Nat. Commun..

[49] van Drunen L., Dobbelaar S., Crone E.A., Wierenga L.M. (2024). Genetic and environmental influences on structural brain development from childhood to adolescence: A longitudinal twin study on cortical thickness, surface area, and subcortical volume. Dev. Cogn. Neurosci..

[50] Kosuge S., Masaoka Y., Kasai H., Honma M., Murakami K., Yoshii N., Watanabe K., Naito T., Kosuge M., Matsui M. (2024). The right amygdala and migraine: Analyzing volume reduction and its relationship with symptom severity. PLoS ONE.

[51] Ou Y., Ni X., Gao X., Yu Y., Zhang Y., Wang Y., Liu J., Yin Z., Rong J., Sun M. (2024). Structural and functional changes of anterior cingulate cortex subregions in migraine without aura: Relationships with pain sensation and pain emotion. Cereb. Cortex.

[52] Younis S., Hougaard A., Noseda R., Ashina M. (2019). Current understanding of thalamic structure and function in migraine. Cephalalgia.

[53] Schmitz N., Admiraal-Behloul F., Arkink E.B., Kruit M.C., Schoonman G.G., Ferrari M.D., van Buchem M.A. (2008). Attack frequency and disease duration as indicators for brain damage in migraine. Headache.

[54] Rocca M.A., Ceccarelli A., Falini A., Colombo B., Tortorella P., Bernasconi L., Comi G., Scotti G., Filippi M. (2006). Brain gray matter changes in migraine patients with T2-visible lesions: A 3-T MRI study. Stroke.

[55] Chong C.D., Schwedt T.J. (2015). Migraine affects white-matter tract integrity: A diffusion-tensor imaging study. Cephalalgia.

[56] Zhang J., Su J., Wang M., Zhao Y., Yao Q., Zhang Q., Lu H., Zhang H., Wang S., Li G.-F. (2016). Increased default mode network connectivity and increased regional homogeneity in migraineurs without aura. J. Headache Pain.

[57] Tessitore A., Russo A., Giordano A., Conte F., Corbo D., De Stefano M., Cirillo S., Cirillo M., Esposito F., Tedeschi G. (2013). Disrupted default mode network connectivity in migraine without aura. J. Headache Pain.

[58] Coppola G., Di Renzo A., Tinelli E., Di Lorenzo C., Scapeccia M., Parisi V., Serrao M., Evangelista M., Ambrosini A., Colonnese C. (2018). Resting state connectivity between default mode network and insula encodes acute migraine headache. Cephalalgia.

[59] Martinelli D., Castellazzi G., De Icco R., Bacila A., Allena M., Faggioli A., Sances G., Pichiecchio A., Borsook D., Wheeler-Kingshott C.A.M.G. (2021). Thalamocortical connectivity in experimentally induced migraine attacks: A pilot study. Brain Sci..

[60] Shin K.J., Lee H.J., Park K.M. (2019). Alterations of individual thalamic nuclei volumes in patients with migraine. J. Headache Pain.

[61] Giardina I., Di Renzo A., Chiffi D., Giuliani G., Sebastianelli G., Casillo F., Abagnale C., Ziccardi L., Pucci A., Parisi V. (2025). 3T MRI thalamic segmentation reveals no macrostructural changes in interictal episodic migraine without aura compared to healthy controls. J. Headache Pain.

