# Supplementary material for: Multiplatform Morphometric Profiling of Whole-Brain, Cerebellar Subregional, and Thalamic Nuclei Alterations in Pediatric Migraine Without Aura
Source: Diagnostics (Basel). 2026 Jul 3;16(13):2085. doi: 10.3390/diagnostics16132085 (PMC13360080; doi:10.3390/diagnostics16132085)
Supplement: Supplementary file 1 [file diagnostics-16-02085-s001.zip › Supplementary Material S1.pdf]

For research only

vol2Brain volumetry report

version 1.0 release 23-11-2021.

Subject: job1991558

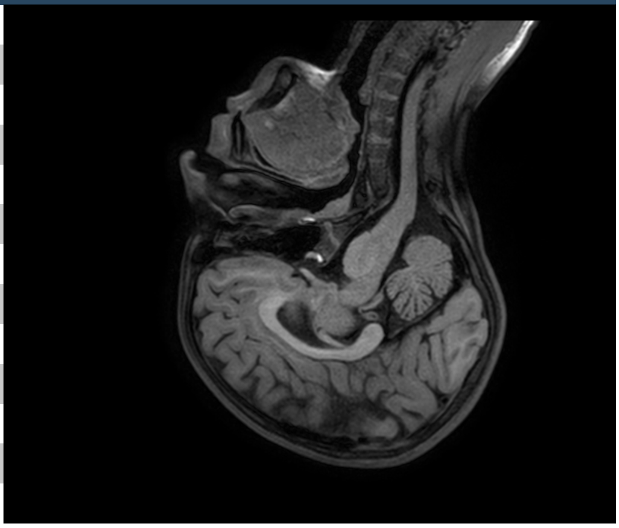

**Sex**	
Male	
**Age**	
13	
**Report date**	
26-May-2026	
**Image orientation**	
neurological	
**Scale factor**	
0.71	
**SNR**	
77.30	

Tissue segmentation

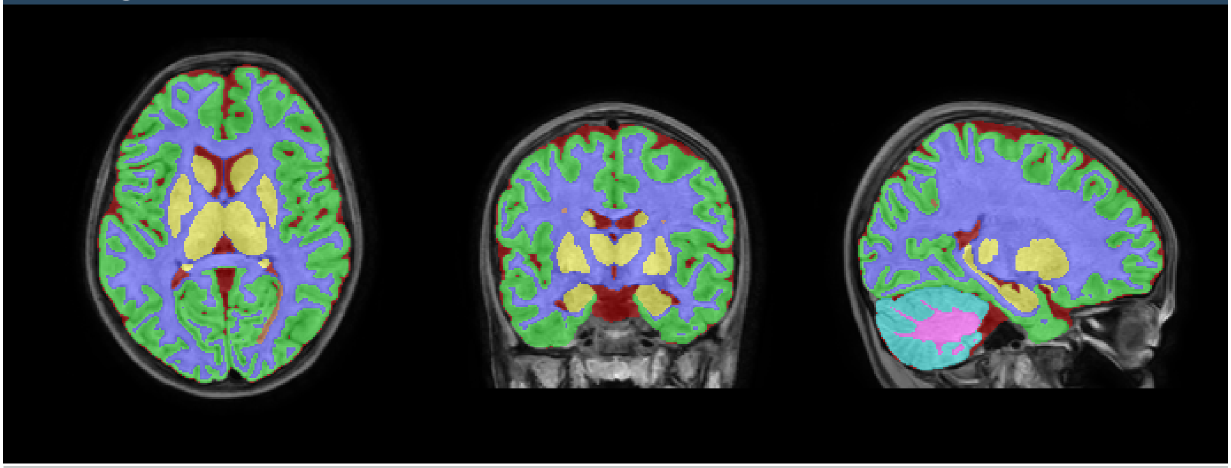

Tissue	Volume (cm^3/%)	
**White Matter (WM)**	432.16 (34.136)	[31.387, 40.773]
Normal Appearing White Matter	430.46 (34.002)	[31.308, 40.703]
Abnormal Appearing White Matter	1.69 (0.134)	[0.000, 0.270]
**Grey Matter (GM)**	685.76 (54.168)	[49.403, 56.960]
Subcortical Grey Matter	41.99 (3.317)	[2.830, 3.519]
Cortical Grey Matter	539.95 (42.651)	[38.613, 45.158]
Cerebellar Grey Matter	103.82 (8.200)	[6.977, 9.267]
**Cerebro Spinal Fluid (CSF)**	129.78 (10.251)	[4.830, 13.854]
**Brain (WM+GM)**	1117.91 (88.304)	[84.789, 93.734]
**Intracranial Cavity (IC)**	1265.99 (100.000)	[100.000, 100.000]

**All the volumes are presented in absolute value (measured in cm^3) and in relative value (measured in relation to the ICV).*

**The Asymmetry Index is calculated as the difference between right and left volumes divided by their mean (in percent).*

**Segmentation images are located in the MNI space (neurological orientation).*

**Values between brackets show expected limits (95%) of normalized volume in function of sex and age for each measure for reference purpose. Values outside the limits are highlighted in red.*

Macrostructures

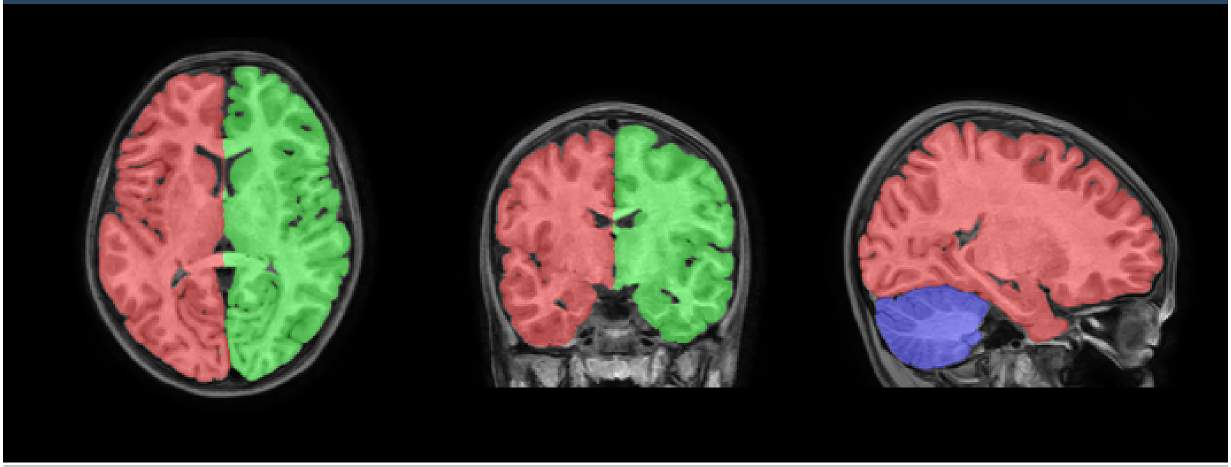

Structure	Total (cm^3/%)	Right (cm^3/%)	Left (cm^3/%)	Asymmetry (%)
**Cerebrum**	989.27 (78.142) [74.434, 83.380]	494.56 (39.065) [37.103, 41.681]	494.70 (39.076) [37.289, 41.742]	-0.0277 [-1.965, 1.321]
Cerebrum WM	407.33 (32.174) [29.389, 38.306]	203.10 (16.043) [14.631, 19.207]	204.23 (16.132) [14.740, 19.117]	-0.5558 [-2.386, 2.257]
Cerebrum GM	581.94 (45.967) [41.655, 48.464]	291.47 (23.023) [20.780, 24.166]	290.47 (22.944) [20.861, 24.313]	0.3419 [-2.096, 0.982]
**Cerebellum ***	120.16 (9.491) [8.403, 10.924]	60.48 (4.778) [4.199, 5.492]	59.68 (4.714) [4.193, 5.442]	1.3460 [-2.929, 4.100]
Cerebellum WM	24.83 (1.962) [1.737, 2.728]	12.67 (1.001) [0.867, 1.377]	12.16 (0.961) [0.865, 1.355]	4.0693 [-4.683, 6.802]
Cerebellum GM	103.82 (8.200) [6.977, 9.267]	47.82 (3.777) [3.177, 4.271]	47.51 (3.753) [3.183, 4.231]	0.6366 [-3.656, 4.640]
**Vermis**	8.49 (0.671) [0.561, 0.821]			
**Brainstem**	18.30 (1.445) [1.194, 1.599]			

**Cerebellum volumes does not include vermis volume.*

Structure segmentation

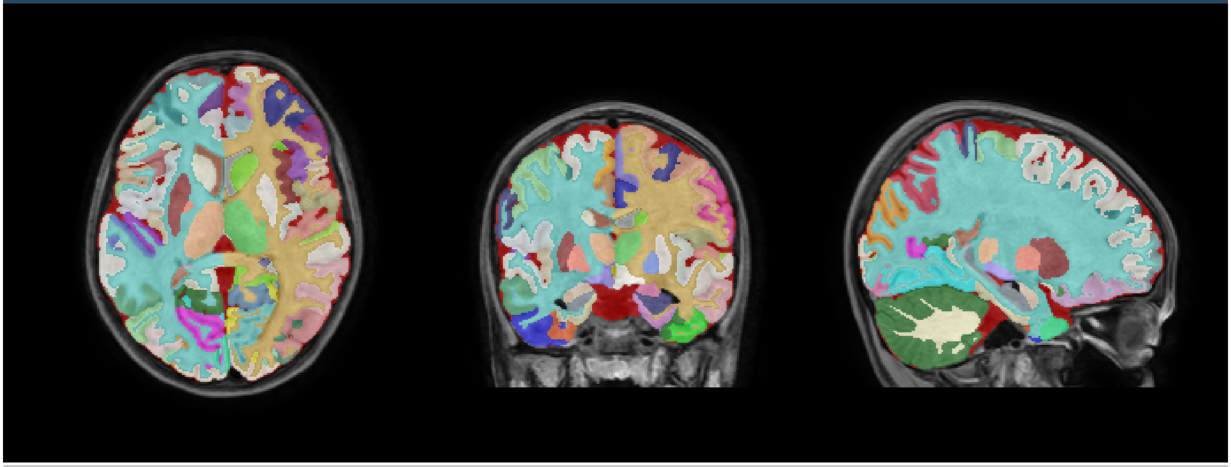

Subcortical	Total (cm^3/%)	Right (cm^3/%)	Left (cm^3/%)	Asymmetry (%)
Accumbens	0.65 (0.051)<br>[0.035, 0.066]	0.34 (0.027)<br>[0.016, 0.032]	0.31 (0.024)<br>[0.018, 0.035]	11.3786<br>[-39.593, 18.507]
Amygdala	1.63 (0.129)<br>[0.110, 0.168]	0.84 (0.066)<br>[0.055, 0.084]	0.79 (0.063)<br>[0.054, 0.086]	5.8337<br>[-15.261, 14.077]
Basal Forebrain	0.68 (0.053)<br>[0.039, 0.069]	0.32 (0.025)<br>[0.019, 0.036]	0.35 (0.028)<br>[0.018, 0.036]	-9.4637<br>[-36.496, 32.006]
Caudate	8.61 (0.680)<br>[0.465, 0.688]	4.37 (0.345)<br>[0.233, 0.344]	4.24 (0.335)<br>[0.230, 0.346]	2.8397<br>[-8.399, 8.040]
Hippocampus	6.81 (0.538)<br>[0.439, 0.639]	3.44 (0.272)<br>[0.215, 0.322]	3.37 (0.266)<br>[0.220, 0.321]	1.9624<br>[-11.879, 10.230]
Pallidum	2.83 (0.224)<br>[0.178, 0.262]	1.34 (0.106)<br>[0.089, 0.132]	1.49 (0.118)<br>[0.088, 0.132]	-10.7322<br>[-12.135, 11.118]
Putamen	8.27 (0.653)<br>[0.577, 0.810]	4.03 (0.318)<br>[0.286, 0.401]	4.25 (0.335)<br>[0.289, 0.411]	-5.3432<br>[-9.923, 6.209]
Thalamus	12.51 (0.988)<br>[0.780, 1.022]	6.23 (0.492)<br>[0.385, 0.505]	6.28 (0.496)<br>[0.391, 0.519]	-0.8295<br>[-9.155, 4.414]
Ventral DC	8.27 (0.653)<br>[0.646, 0.819]	4.22 (0.333)<br>[0.316, 0.405]	4.05 (0.320)<br>[0.328, 0.416]	4.1427<br>[-7.838, 1.580]

Cortical	Total (cm^3/%)	Right (cm^3/%)	Left (cm^3/%)	Asymmetry (%)
**Frontal lobe**	182.96 (14.452) [12.984, 15.522]	92.51 (7.307) [6.443, 7.758]	90.45 (7.145) [6.509, 7.796]	2.2496 [-5.242, 3.922]
Frontal pole	6.98 (0.552) [0.386, 0.628]	3.56 (0.281) [0.195, 0.331]	3.43 (0.271) [0.186, 0.326]	3.7444 [-27.080, 22.422]
Gyrus rectus	4.16 (0.328) [0.220, 0.363]	1.97 (0.156) [0.109, 0.194]	2.19 (0.173) [0.101, 0.179]	-10.3607 [-23.385, 37.614]
Opercular inf. frontal gyrus	8.00 (0.632) [0.421, 0.667]	3.73 (0.295) [0.186, 0.341]	4.27 (0.338) [0.202, 0.359]	-13.5867 [-48.578, 34.123]
Orbital inf. frontal gyrus	4.08 (0.322) [0.150, 0.325]	1.82 (0.144) [0.076, 0.180]	2.26 (0.178) [0.049, 0.170]	-21.4634 [-59.391, 96.584]
Triangular inf. frontal gyrus	7.09 (0.560) [0.441, 0.690]	3.16 (0.249) [0.206, 0.370]	3.93 (0.310) [0.201, 0.355]	-21.7452 [-37.000, 42.540]
Medial frontal cortex	2.42 (0.191) [0.198, 0.333]	1.20 (0.095) [0.090, 0.183]	1.22 (0.096) [0.087, 0.170]	-1.7064 [-42.459, 58.467]
Middle frontal gyrus	42.57 (3.362) [2.855, 3.643]	23.26 (1.837) [1.414, 1.859]	19.31 (1.525) [1.404, 1.821]	18.5649 [-10.996, 13.891]
Anterior orbital gyrus	4.80 (0.379) [0.225, 0.378]	2.93 (0.231) [0.110, 0.203]	1.87 (0.148) [0.099, 0.191]	44.2436 [-33.991, 46.510]
Lateral orbital gyrus	4.53 (0.358) [0.239, 0.439]	2.35 (0.185) [0.113, 0.230]	2.18 (0.172) [0.107, 0.228]	7.3121 [-41.179, 46.699]
Medial orbital gyrus	8.22 (0.649) [0.402, 0.637]	4.15 (0.328) [0.234, 0.354]	4.07 (0.321) [0.193, 0.329]	2.0579 [-25.370, 16.022]
Posterior orbital gyrus	5.78 (0.457) [0.356, 0.575]	2.99 (0.236) [0.156, 0.285]	2.79 (0.220) [0.184, 0.305]	7.0270 [-40.098, 20.093]
Precentral gyrus	24.92 (1.969) [1.753, 2.301]	12.52 (0.989) [0.857, 1.175]	12.40 (0.980) [0.862, 1.160]	0.9523 [-14.927, 16.287]
Precentral gyrus medial segment	5.35 (0.422) [0.319, 0.485]	2.58 (0.203) [0.157, 0.259]	2.77 (0.219) [0.143, 0.246]	-7.3109 [-24.954, 37.047]
Subcallosal area	2.12 (0.168) [0.074, 0.221]	1.12 (0.088) [0.037, 0.115]	1.00 (0.079) [0.034, 0.109]	11.1260 [-17.640, 26.542]
Sup. frontal gyrus	29.34 (2.317) [2.060, 2.749]	13.28 (1.049) [1.016, 1.387]	16.06 (1.268) [1.009, 1.397]	-18.9226 [-15.529, 15.836]
Sup. frontal gyrus medial segment	12.50 (0.988) [0.885, 1.247]	6.62 (0.523) [0.417, 0.655]	5.88 (0.465) [0.417, 0.643]	11.7420 [-32.335, 31.789]
Supplementary motor cortex	10.12 (0.799) [0.649, 0.951]	5.28 (0.417) [0.310, 0.487]	4.83 (0.382) [0.307, 0.496]	8.8936 [-29.540, 28.227]

**Temporal lobe**	104.83 (8.280)<br>[7.060, 8.737]	52.65 (4.159)<br>[3.526, 4.393]	52.17 (4.121)<br>[3.501, 4.378]	0.9220<br>[-6.298, 7.452]
Fusiform gyrus	14.18 (1.120)<br>[0.970, 1.445]	7.09 (0.560)<br>[0.474, 0.746]	7.09 (0.560)<br>[0.466, 0.729]	-0.0501<br>[-32.845, 14.966]
Planum polare	3.09 (0.244)<br>[0.253, 0.370]	1.55 (0.123)<br>[0.117, 0.187]	1.54 (0.122)<br>[0.124, 0.196]	0.7353<br>[-34.639, 22.947]
Planum temporale	4.16 (0.329)<br>[0.239, 0.411]	1.56 (0.123)<br>[0.112, 0.210]	2.60 (0.206)<br>[0.108, 0.220]	-50.1793<br>[-41.981, 44.510]
Inf. temporal gyrus	18.80 (1.485)<br>[1.444, 2.066]	9.05 (0.715)<br>[0.682, 1.026]	9.75 (0.770)<br>[0.719, 1.083]	-7.4388<br>[-26.093, 14.830]
Middle temporal gyrus	28.27 (2.233)<br>[2.036, 2.644]	14.30 (1.129)<br>[0.990, 1.339]	13.97 (1.104)<br>[0.998, 1.353]	2.2827<br>[-19.297, 16.174]
Sup. temporal gyrus	15.70 (1.240)<br>[1.004, 1.433]	7.91 (0.625)<br>[0.529, 0.784]	7.79 (0.615)<br>[0.431, 0.693]	1.6026<br>[-17.943, 34.118]
Transverse temporal gyrus	3.51 (0.277)<br>[0.166, 0.315]	1.88 (0.148)<br>[0.071, 0.153]	1.63 (0.129)<br>[0.083, 0.175]	14.0514<br>[-53.909, 24.215]
Temporal pole	17.11 (1.352)<br>[1.124, 1.706]	9.32 (0.736)<br>[0.587, 0.879]	7.80 (0.616)<br>[0.516, 0.848]	17.8044<br>[-9.571, 25.868]
**Parietal lobe**	110.06 (8.694)<br>[7.855, 9.605]	55.96 (4.420)<br>[3.901, 4.804]	54.11 (4.274)<br>[3.916, 4.837]	3.3639<br>[-7.069, 5.956]
Angular gyrus	21.69 (1.714)<br>[1.317, 2.026]	11.32 (0.894)<br>[0.672, 1.064]	10.38 (0.820)<br>[0.613, 0.994]	8.6347<br>[-14.588, 30.182]
Postcentral gyrus	19.56 (1.545)<br>[1.225, 1.687]	10.02 (0.791)<br>[0.584, 0.859]	9.55 (0.754)<br>[0.604, 0.865]	4.8171<br>[-21.981, 18.317]
Postcentral gyrus medial segment	1.58 (0.125)<br>[0.083, 0.170]	0.69 (0.054)<br>[0.036, 0.092]	0.89 (0.071)<br>[0.034, 0.090]	-26.0791<br>[-55.688, 58.578]
Precuneus	24.92 (1.969)<br>[1.461, 2.008]	13.12 (1.036)<br>[0.711, 1.016]	11.80 (0.932)<br>[0.728, 1.014]	10.5497<br>[-15.092, 13.094]
Sup. parietal lobule	23.89 (1.887)<br>[1.241, 1.745]	11.34 (0.895)<br>[0.583, 0.873]	12.55 (0.991)<br>[0.622, 0.908]	-10.1737<br>[-24.944, 15.560]
Supramarginal gyrus	18.42 (1.455)<br>[1.199, 1.636]	9.48 (0.749)<br>[0.568, 0.821]	8.93 (0.706)<br>[0.591, 0.855]	5.9736<br>[-28.915, 19.624]

**Occipital lobe**	71.37 (5.637) [4.586, 6.099]	34.09 (2.693) [2.298, 3.120]	37.27 (2.944) [2.221, 3.046]	-8.9241 [-9.553, 15.567]
Calcarine cortex	7.67 (0.606) [0.283, 0.598]	3.42 (0.270) [0.139, 0.306]	4.25 (0.336) [0.132, 0.303]	-21.8269 [-25.041, 30.937]
Cuneus	6.65 (0.526) [0.493, 0.787]	3.12 (0.246) [0.230, 0.400]	3.54 (0.279) [0.238, 0.412]	-12.6469 [-33.074, 26.974]
Lingual gyrus	17.40 (1.375) [0.988, 1.414]	8.48 (0.670) [0.475, 0.710]	8.92 (0.705) [0.483, 0.733]	-5.0637 [-21.632, 17.180]
Occipital fusiform gyrus	6.12 (0.483) [0.348, 0.627]	3.14 (0.248) [0.162, 0.324]	2.98 (0.235) [0.161, 0.328]	5.1365 [-37.974, 34.646]
Inf. occipital gyrus	11.84 (0.935) [0.759, 1.128]	6.06 (0.479) [0.360, 0.582]	5.77 (0.456) [0.363, 0.582]	4.9226 [-27.419, 28.404]
Middle occipital gyrus	10.78 (0.852) [0.608, 0.993]	4.77 (0.377) [0.269, 0.469]	6.01 (0.475) [0.313, 0.550]	-23.0835 [-45.514, 12.925]
Sup. occipital gyrus	7.43 (0.587) [0.356, 0.673]	3.54 (0.280) [0.207, 0.391]	3.89 (0.307) [0.155, 0.321]	-9.4509 [-19.377, 46.987]
Occipital pole	3.48 (0.275) [0.183, 0.460]	1.57 (0.124) [0.077, 0.222]	1.91 (0.151) [0.087, 0.257]	-19.4972 [-60.891, 30.946]
**Limbic cortex**	40.76 (3.219) [2.860, 3.698]	20.97 (1.656) [1.421, 1.875]	19.79 (1.563) [1.389, 1.872]	5.7679 [-13.422, 15.316]
Entorhinal area	3.19 (0.252) [0.228, 0.357]	1.67 (0.132) [0.111, 0.186]	1.52 (0.120) [0.107, 0.182]	9.5365 [-23.309, 28.073]
Anterior cingulate gyrus	10.26 (0.810) [0.763, 1.170]	5.80 (0.458) [0.352, 0.610]	4.46 (0.352) [0.371, 0.600]	26.2797 [-34.722, 32.695]
Middle cingulate gyrus	11.54 (0.912) [0.703, 0.987]	5.90 (0.466) [0.342, 0.508]	5.64 (0.445) [0.332, 0.509]	4.6179 [-24.037, 27.048]
Posterior cingulate gyrus	10.45 (0.825) [0.616, 0.868]	5.10 (0.403) [0.291, 0.440]	5.35 (0.422) [0.310, 0.443]	-4.7334 [-22.405, 15.868]
Parahippocampal gyrus	5.32 (0.420) [0.345, 0.519]	2.49 (0.196) [0.167, 0.272]	2.83 (0.224) [0.173, 0.269]	-12.9377 [-22.698, 13.852]
**Insular cortex**	29.98 (2.368) [2.088, 2.677]	14.35 (1.134) [1.061, 1.378]	15.62 (1.234) [1.011, 1.315]	-8.4681 [-4.816, 14.092]
Anterior insula	8.66 (0.684) [0.563, 0.798]	4.43 (0.350) [0.272, 0.396]	4.23 (0.334) [0.286, 0.407]	4.6466 [-15.197, 7.886]
Posterior insula	4.30 (0.340) [0.283, 0.424]	2.02 (0.159) [0.143, 0.221]	2.28 (0.180) [0.134, 0.208]	-12.3906 [-11.526, 23.507]
Central operculum	7.99 (0.631) [0.540, 0.738]	3.76 (0.297) [0.262, 0.378]	4.23 (0.334) [0.263, 0.375]	-11.7396 [-19.748, 20.810]
Frontal operculum	3.95 (0.312) [0.264, 0.415]	1.80 (0.142) [0.123, 0.206]	2.15 (0.170) [0.127, 0.224]	-17.6778 [-62.103, 14.905]
Parietal operculum	5.08 (0.401) [0.275, 0.464]	2.35 (0.185) [0.109, 0.215]	2.73 (0.216) [0.150, 0.265]	-15.1909 [-62.025, 13.782]

CSF	Total (cm^3/%)	Right (cm^3/%)	Left (cm^3/%)	Asymmetry (%)
Inf. Lateral Ventricle	0.85 (0.067) [0.005, 0.096]	0.37 (0.029) [0.000, 0.055]	0.48 (0.038) [0.002, 0.046]	-26.0434 [-110.5, 104.8]
Lateral Ventricle	9.17 (0.724) [0.000, 2.545]	4.08 (0.322) [0.000, 1.324]	5.09 (0.402) [0.000, 1.278]	-22.1809 [-55.050, 56.137]
3rd Ventricle	0.78 (0.061) [0.000, 0.120]			
4th Ventricle	1.88 (0.148) [0.054, 0.213]			
External CSF	117.10 (9.250) [3.568, 12.205]			

Cerebellar vermis	Total (cm^3/%)
Lobules I-V	3.93 (0.311) [0.235, 0.393]
Lobules VI-VII	1.76 (0.139) [0.122, 0.203]
Lobules VIII-X	2.79 (0.221) [0.167, 0.263]

Cortical thickness

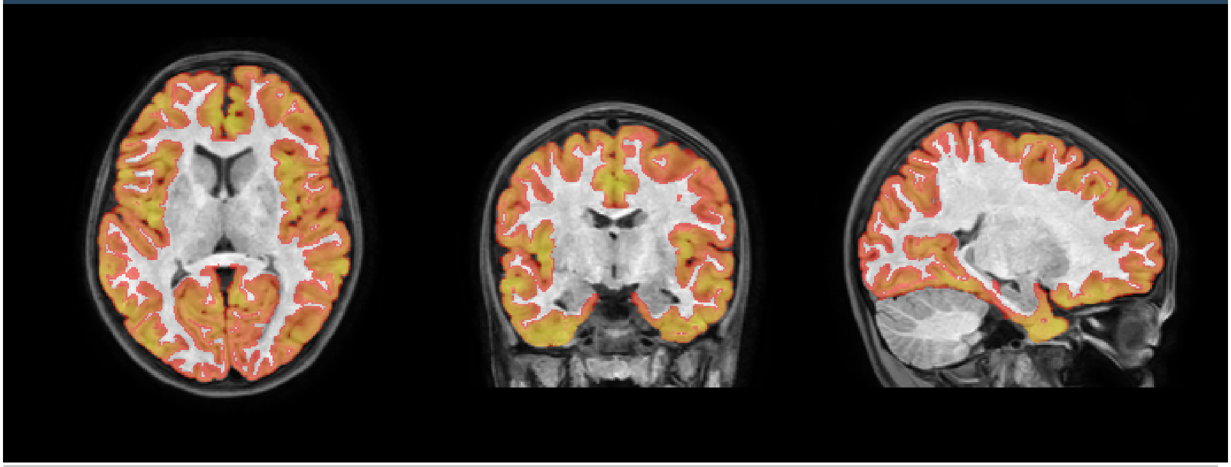

Thickness	Total (mm/norm.)	Right (mm/norm.)	Left (mm/norm.)	Asymmetry (%)
**Frontal lobe**	2.85 (0.026) [0.021, 0.029]	2.93 (0.027) [0.021, 0.029]	2.76 (0.026) [0.021, 0.029]	5.9269 [-7.123, 5.915]
Frontal pole	2.55 (0.024) [0.021, 0.031]	2.86 (0.026) [0.021, 0.032]	2.22 (0.020) [0.019, 0.031]	25.5534 [-11.157, 35.141]
Gyrus rectus	4.40 (0.041) [0.020, 0.038]	4.45 (0.041) [0.020, 0.039]	4.34 (0.040) [0.011, 0.033]	2.5076 [-23.678, 31.831]
Opercular inf. frontal gyrus	2.45 (0.023) [0.019, 0.027]	2.14 (0.020) [0.018, 0.027]	2.71 (0.025) [0.019, 0.027]	-23.2368 [-27.078, 20.646]
Orbital inf. frontal gyrus	2.84 (0.026) [0.020, 0.030]	2.90 (0.027) [0.021, 0.032]	2.79 (0.026) [0.019, 0.029]	3.9188 [-16.026, 34.661]
Triangular inf. frontal gyrus	2.62 (0.024) [0.020, 0.028]	2.54 (0.023) [0.019, 0.029]	2.69 (0.025) [0.019, 0.028]	-5.6939 [-21.830, 23.428]
Medial frontal cortex	3.41 (0.031) [0.024, 0.035]	3.72 (0.034) [0.025, 0.037]	3.10 (0.029) [0.023, 0.035]	18.1299 [-15.405, 28.117]
Middle frontal gyrus	2.90 (0.027) [0.021, 0.031]	2.89 (0.027) [0.021, 0.032]	2.92 (0.027) [0.021, 0.032]	-1.2075 [-15.168, 13.588]
Anterior orbital gyrus	3.75 (0.035) [0.024, 0.036]	3.92 (0.036) [0.023, 0.037]	3.47 (0.032) [0.023, 0.037]	12.1897 [-22.928, 20.639]
Lateral orbital gyrus	3.46 (0.032) [0.023, 0.034]	3.57 (0.033) [0.023, 0.036]	3.35 (0.031) [0.021, 0.033]	6.5109 [-10.203, 31.618]
Medial orbital gyrus	3.48 (0.032) [0.020, 0.034]	3.23 (0.030) [0.018, 0.034]	3.73 (0.034) [0.021, 0.034]	-14.3482 [-25.872, 11.563]
Posterior orbital gyrus	2.82 (0.026) [0.024, 0.037]	3.05 (0.028) [0.024, 0.038]	2.58 (0.024) [0.023, 0.037]	16.6624 [-15.192, 19.704]
Precentral gyrus	1.94 (0.018) [0.015, 0.022]	1.95 (0.018) [0.015, 0.022]	1.94 (0.018) [0.015, 0.022]	0.6033 [-17.589, 12.124]
Precentral gyrus medial segment	2.28 (0.021) [0.014, 0.023]	2.47 (0.023) [0.015, 0.024]	2.11 (0.019) [0.014, 0.023]	15.8282 [-14.984, 30.585]
Subcallosal area	3.44 (0.032) [0.012, 0.032]	3.62 (0.034) [0.011, 0.032]	3.24 (0.030) [0.013, 0.033]	11.3663 [-38.610, 26.487]

Sup. frontal gyrus	2.71 (0.025) [0.020, 0.028]	2.82 (0.026) [0.019, 0.028]	2.63 (0.024) [0.020, 0.028]	6.9846 [-10.848, 10.302]
Sup. frontal gyrus medial segment	3.43 (0.032) [0.025, 0.034]	3.79 (0.035) [0.025, 0.035]	3.02 (0.028) [0.024, 0.034]	22.7873 [-13.763, 18.474]
Supplementary motor cortex	3.42 (0.032) [0.021, 0.031]	3.71 (0.034) [0.021, 0.031]	3.10 (0.029) [0.020, 0.030]	17.7281 [-11.149, 22.188]
**Temporal lobe**	3.33 (0.031) [0.016, 0.024]	3.38 (0.031) [0.017, 0.024]	3.28 (0.030) [0.016, 0.023]	2.9880 [-5.027, 13.004]
Fusiform gyrus	3.46 (0.032) [0.027, 0.039]	3.37 (0.031) [0.026, 0.039]	3.56 (0.033) [0.027, 0.039]	-5.5726 [-12.531, 9.055]
Planum polare	1.73 (0.016) [0.015, 0.025]	1.96 (0.018) [0.015, 0.026]	1.50 (0.014) [0.014, 0.026]	26.4014 [-23.111, 31.452]
Planum temporale	2.26 (0.021) [0.017, 0.026]	2.48 (0.023) [0.016, 0.026]	2.12 (0.020) [0.016, 0.027]	15.5888 [-47.554, 14.721]
Inf. temporal gyrus	3.65 (0.034) [0.026, 0.037]	3.66 (0.034) [0.024, 0.036]	3.63 (0.034) [0.028, 0.039]	0.9123 [-21.441, 0.535]
Middle temporal gyrus	3.45 (0.032) [0.025, 0.034]	3.44 (0.032) [0.024, 0.034]	3.47 (0.032) [0.024, 0.034]	-0.8912 [-10.024, 12.364]
Sup. temporal gyrus	2.75 (0.025) [0.020, 0.028]	2.75 (0.025) [0.021, 0.029]	2.74 (0.025) [0.019, 0.028]	0.4435 [-12.833, 20.490]
Transverse temporal gyrus	2.38 (0.022) [0.016, 0.027]	2.48 (0.023) [0.016, 0.027]	2.26 (0.021) [0.015, 0.028]	9.2903 [-28.320, 23.735]
Temporal pole	3.95 (0.037) [0.029, 0.041]	4.13 (0.038) [0.029, 0.041]	3.74 (0.035) [0.028, 0.041]	9.7699 [-12.242, 14.591]
**Parietal lobe**	2.49 (0.023) [0.025, 0.034]	2.44 (0.023) [0.025, 0.034]	2.54 (0.023) [0.025, 0.034]	-3.9743 [-5.137, 8.288]
Angular gyrus	2.78 (0.026) [0.019, 0.028]	2.60 (0.024) [0.018, 0.028]	2.98 (0.028) [0.019, 0.029]	-13.4585 [-24.379, 14.042]
Postcentral gyrus	1.71 (0.016) [0.011, 0.016]	1.65 (0.015) [0.010, 0.016]	1.77 (0.016) [0.011, 0.017]	-7.2723 [-23.596, 17.529]
Postcentral gyrus medial segment	1.05 (0.010) [0.006, 0.015]	0.89 (0.008) [0.006, 0.017]	1.17 (0.011) [0.006, 0.015]	-27.1771 [-40.816, 59.137]
Precuneus	3.29 (0.030) [0.019, 0.030]	3.18 (0.029) [0.019, 0.030]	3.42 (0.032) [0.020, 0.031]	-7.0478 [-16.005, 11.106]
Sup. parietal lobule	2.03 (0.019) [0.011, 0.019]	2.13 (0.020) [0.011, 0.019]	1.94 (0.018) [0.012, 0.020]	9.3361 [-28.779, 10.024]
Supramarginal gyrus	2.61 (0.024) [0.019, 0.027]	2.54 (0.024) [0.018, 0.027]	2.67 (0.025) [0.019, 0.028]	-5.0119 [-22.404, 14.674]

**Occipital lobe**	2.38 (0.022) [0.015, 0.025]	2.24 (0.021) [0.015, 0.026]	2.52 (0.023) [0.015, 0.025]	-11.6858 [-8.044, 10.213]
Calcarine cortex	2.03 (0.019) [0.008, 0.022]	1.72 (0.016) [0.008, 0.023]	2.29 (0.021) [0.009, 0.023]	-28.3113 [-31.769, 21.071]
Cuneus	2.08 (0.019) [0.011, 0.022]	2.08 (0.019) [0.010, 0.022]	2.08 (0.019) [0.011, 0.022]	0.0348 [-27.476, 19.688]
Lingual gyrus	2.57 (0.024) [0.017, 0.029]	2.50 (0.023) [0.017, 0.030]	2.63 (0.024) [0.017, 0.029]	-5.3237 [-13.630, 13.587]
Occipital fusiform gyrus	2.50 (0.023) [0.016, 0.028]	2.39 (0.022) [0.016, 0.028]	2.60 (0.024) [0.015, 0.028]	-8.4960 [-18.362, 23.912]
Inf. occipital gyrus	2.44 (0.023) [0.018, 0.028]	2.30 (0.021) [0.017, 0.027]	2.60 (0.024) [0.017, 0.028]	-12.2469 [-19.356, 13.657]
Middle occipital gyrus	3.21 (0.030) [0.018, 0.029]	2.82 (0.026) [0.017, 0.029]	3.51 (0.032) [0.017, 0.030]	-21.8917 [-21.073, 18.795]
Sup. occipital gyrus	1.69 (0.016) [0.011, 0.020]	1.68 (0.016) [0.011, 0.021]	1.70 (0.016) [0.010, 0.020]	-0.6398 [-20.302, 33.932]
Occipital pole	1.35 (0.012) [0.007, 0.019]	1.24 (0.011) [0.006, 0.019]	1.44 (0.013) [0.007, 0.019]	-15.3244 [-34.701, 33.236]
**Limbic cortex**	3.58 (0.033) [0.026, 0.035]	3.70 (0.034) [0.026, 0.035]	3.46 (0.032) [0.026, 0.036]	6.8733 [-9.151, 4.876]
Entorhinal area	3.08 (0.029) [0.026, 0.033]	3.32 (0.031) [0.025, 0.035]	2.82 (0.026) [0.024, 0.034]	16.3236 [-13.605, 21.797]
Anterior cingulate gyrus	4.07 (0.038) [0.030, 0.041]	4.31 (0.040) [0.030, 0.042]	3.75 (0.035) [0.029, 0.041]	13.8672 [-9.069, 14.915]
Middle cingulate gyrus	3.74 (0.035) [0.024, 0.035]	3.81 (0.035) [0.024, 0.036]	3.66 (0.034) [0.023, 0.036]	3.9983 [-12.255, 16.746]
Posterior cingulate gyrus	3.48 (0.032) [0.024, 0.036]	3.46 (0.032) [0.024, 0.036]	3.50 (0.032) [0.025, 0.036]	-1.0967 [-12.309, 10.213]
Parahippocampal gyrus	2.84 (0.026) [0.019, 0.030]	2.81 (0.026) [0.019, 0.030]	2.87 (0.027) [0.019, 0.029]	-2.1738 [-12.109, 19.446]
**Insular cortex**	3.19 (0.030) [0.023, 0.032]	3.20 (0.030) [0.023, 0.033]	3.19 (0.029) [0.023, 0.033]	0.3814 [-8.991, 8.498]
Anterior insula	3.71 (0.034) [0.027, 0.037]	3.91 (0.036) [0.027, 0.037]	3.49 (0.032) [0.027, 0.037]	11.2491 [-9.896, 10.434]
Posterior insula	3.29 (0.030) [0.020, 0.032]	3.14 (0.029) [0.020, 0.033]	3.41 (0.032) [0.019, 0.032]	-8.2112 [-14.410, 20.424]
Central operculum	2.99 (0.028) [0.022, 0.032]	2.91 (0.027) [0.021, 0.032]	3.07 (0.028) [0.022, 0.032]	-5.5557 [-17.353, 14.476]
Frontal operculum	2.90 (0.027) [0.024, 0.035]	2.89 (0.027) [0.023, 0.035]	2.91 (0.027) [0.023, 0.035]	-0.4602 [-33.150, 5.339]
Parietal operculum	2.78 (0.026) [0.017, 0.027]	2.61 (0.024) [0.017, 0.027]	2.92 (0.027) [0.017, 0.028]	-11.3024 [-26.192, 23.099]
