# Supplementary material for: Multiplatform Morphometric Profiling of Whole-Brain, Cerebellar Subregional, and Thalamic Nuclei Alterations in Pediatric Migraine Without Aura
Source: Diagnostics (Basel). 2026 Jul 3;16(13):2085. doi: 10.3390/diagnostics16132085 (PMC13360080; doi:10.3390/diagnostics16132085)

CERES volumetry report

For research only

version 1.0 release 03-10-2018.

Patient ID	Sex	Age	Report Date
job1991559	Male	13	26-May-2026

Image Information	
Orientation	neurological
Scale factor	0.73
SNR	499.19
Total intracranial volume (cm^3)	1308.88

Volumes[1]	Total (cm^3/%)	Right (cm^3/%)	Left (cm^3/%)	Asym.(%)[2]
Cerebellum	123.17 (9.4106)	61.94 (4.7322)	61.24 (4.6784)	1.1416
	[8.0988, 10.7590][5]	[4.0544, 5.3952]	[4.0333, 5.3749]	[-3.2930, 4.3518]
Lobule I-II	0.11 (0.0085)	0.06 (0.0048)	0.05 (0.0037)	24.8366
	[0.0028, 0.0265]	[0.0023, 0.0128]	[0.0001, 0.0142]	[-35.3979, 55.0613]
Lobule III	1.34 (0.1027)	0.73 (0.0555)	0.62 (0.0472)	16.1693
	[0.0757, 0.1622]	[0.0362, 0.0815]	[0.0372, 0.0830]	[-25.4545, 23.5096]
Lobule IV	5.59 (0.4274)	2.72 (0.2077)	2.88 (0.2198)	-5.6577
	[0.2530, 0.4297]	[0.1177, 0.2190]	[0.1259, 0.2201]	[-29.8665, 23.7181]
Lobule V	8.04 (0.6146)	4.03 (0.3077)	4.02 (0.3069)	0.2901
	[0.2530, 0.4297]	[0.1177, 0.2190]	[0.1259, 0.2201]	[-29.8665, 23.7181]
Lobule VI	15.46 (1.1812)	7.85 (0.5997)	7.61 (0.5815)	3.0758
	[1.0256, 1.6461]	[0.4773, 0.8111]	[0.5336, 0.8497]	[-23.1863, 8.7545]
Lobule Crus I	26.72 (2.0413)	13.49 (1.0305)	13.23 (1.0108)	1.9326
	[1.4562, 2.4145]	[0.7416, 1.2339]	[0.6931, 1.2021]	[-11.4980, 19.9597]
Lobule Crus II	17.51 (1.3381)	8.89 (0.6790)	8.63 (0.6591)	2.9816
	[0.8389, 1.5344]	[0.4157, 0.7989]	[0.4033, 0.7554]	[-16.2421, 26.1227]
Lobule VIIB	10.03 (0.7665)	5.05 (0.3856)	4.99 (0.3810)	1.2067
	[0.5235, 0.9164]	[0.2739, 0.4976]	[0.2282, 0.4403]	[-12.8567, 45.1275]
Lobule VIIIA	10.27 (0.7845)	5.07 (0.3874)	5.20 (0.3972)	-2.5000
	[0.7399, 1.1582]	[0.3280, 0.5704]	[0.3869, 0.6128]	[-35.3638, 13.9582]
Lobule VIIIB	7.21 (0.5511)	3.73 (0.2852)	3.48 (0.2659)	6.9970
	[0.4480, 0.7991]	[0.2019, 0.4023]	[0.2235, 0.4193]	[-34.6542, 23.5987]
Lobule IX	6.13 (0.4687)	2.97 (0.2266)	3.17 (0.2421)	-6.6096
	[0.3568, 0.7126]	[0.1775, 0.3598]	[0.1760, 0.3562]	[-12.7177, 14.0034]
Lobule X	1.22 (0.0932)	0.64 (0.0491)	0.58 (0.0441)	10.7656
	[0.3568, 0.7126]	[0.1775, 0.3598]	[0.1760, 0.3562]	[-12.7177, 14.0034]

[1] *All the volumes are presented in absolute value (measured in cm^3) and in relative value (measured in relation to the ICV).*

[2] *The Asymmetry Index is calculated as the difference between right and left volumes divided by their mean (in percent).*

[3] *Cortical thickness is given in absolute value (mm) and also normalized in relation to the cube root of the intracranial volume (adimensional).*

[4] *Result images located in the MNI space (neurological orientation).*

[5] *Values between brackets show expected limits (95%) of normalized volume in function of sex and age for each measure for reference purpose. Values outside the limits are highlighted in red.*

Grey matter vol.	Total (cm^3/%)	Right (cm^3/%)	Left (cm^3/%)	Asym.(%)
Cerebellum	97.08 (7.4171)	48.95 (3.7396)	48.14 (3.6776)	1.6707
	[6.2766, 8.4851]	[3.1201, 4.2649]	[3.1465, 4.2302]	[-4.2157, 4.6435]
Lobule I-II	0.10 (0.0074)	0.05 (0.0040)	0.04 (0.0034)	20.7750
	[0.0020, 0.0127]	[0.0014, 0.0061]	[0.0004, 0.0069]	[-60.8434, 73.3535]
Lobule III	1.19 (0.0913)	0.67 (0.0508)	0.53 (0.0405)	31.1397
	[0.0404, 0.0997]	[0.0188, 0.0495]	[0.0199, 0.0520]	[-44.2728, 35.3296]
Lobule IV	5.21 (0.3978)	2.53 (0.1933)	2.68 (0.2045)	-7.7210
	[0.2182, 0.3720]	[0.1040, 0.1887]	[0.1057, 0.1918]	[-38.0053, 35.4833]
Lobule V	6.97 (0.5324)	3.46 (0.2646)	3.51 (0.2678)	-1.6648
	[0.2182, 0.3720]	[0.1040, 0.1887]	[0.1057, 0.1918]	[-38.0053, 35.4833]
Lobule VI	14.01 (1.0704)	6.99 (0.5339)	7.02 (0.5365)	-0.6709
	[0.9157, 1.4846]	[0.4238, 0.7338]	[0.4780, 0.7647]	[-32.8166, 13.0050]
Lobule Crus I	22.95 (1.7533)	11.73 (0.8959)	11.22 (0.8574)	6.0134
	[1.2366, 2.0944]	[0.6264, 1.0762]	[0.5913, 1.0371]	[-16.4716, 28.0227]
Lobule Crus II	14.88 (1.1367)	7.52 (0.5748)	7.36 (0.5619)	3.1051
	[0.7497, 1.3658]	[0.3711, 0.7085]	[0.3597, 0.6762]	[-23.7689, 35.4986]
Lobule VIIB	9.07 (0.6931)	4.65 (0.3549)	4.43 (0.3382)	6.6359
	[0.4734, 0.8267]	[0.2498, 0.4524]	[0.2040, 0.3939]	[-16.9575, 61.4936]
Lobule VIIIA	9.42 (0.7194)	4.63 (0.3537)	4.79 (0.3657)	-4.5666
	[0.6679, 1.0398]	[0.2954, 0.5100]	[0.3499, 0.5524]	[-48.4122, 18.0760]
Lobule VIIIB	6.35 (0.4854)	3.28 (0.2504)	3.08 (0.2350)	8.6573
	[0.3833, 0.6947]	[0.1702, 0.3459]	[0.1925, 0.3694]	[-50.7570, 29.6383]
Lobule IX	5.33 (0.4076)	2.62 (0.1999)	2.72 (0.2077)	-5.2859
	[0.3015, 0.6045]	[0.1452, 0.3036]	[0.1527, 0.3044]	[-26.2909, 17.6801]
Lobule X	1.18 (0.0900)	0.61 (0.0466)	0.57 (0.0434)	10.0183
	[0.3015, 0.6045]	[0.1452, 0.3036]	[0.1527, 0.3044]	[-26.2909, 17.6801]

**Cortical thickness**[3]	**Mean** $(mm/norm.)$	**Right** $(mm/norm.)$	**Left** $(mm/norm.)$	**Asym.**(%)
Cerebellum	4.92 (4.495)	4.95 (4.521)	4.89 (4.468)	-1.1652
	[4.074, 4.748]	[4.045, 4.768]	[4.085, 4.749]	[-0.0707, 0.0796]
Lobule I-II	3.83 (3.501)	4.02 (3.677)	3.58 (3.277)	-11.4278
	[0.345, 2.314]	[0.384, 2.333]	[0.293, 2.317]	[-0.6491, 0.4558]
Lobule III	4.10 (3.751)	4.17 (3.810)	4.02 (3.678)	-3.5152
	[2.310, 3.983]	[2.288, 4.068]	[2.250, 3.966]	[-0.4071, 0.3014]
Lobule IV	5.26 (4.806)	5.15 (4.704)	5.36 (4.897)	4.0145
	[3.952, 4.986]	[3.951, 5.049]	[3.911, 4.969]	[-0.1675, 0.1136]
Lobule V	5.15 (4.707)	5.16 (4.715)	5.14 (4.699)	-0.3277
	[3.952, 4.986]	[3.951, 5.049]	[3.911, 4.969]	[-0.1675, 0.1136]
Lobule VI	5.04 (4.610)	4.99 (4.558)	5.10 (4.663)	2.2724
	[4.166, 5.005]	[4.117, 5.033]	[4.179, 5.012]	[-0.1025, 0.1204]
Lobule Crus I	4.98 (4.553)	5.07 (4.631)	4.89 (4.472)	-3.5050
	[3.905, 4.780]	[3.854, 4.849]	[3.902, 4.772]	[-0.1595, 0.1457]
Lobule Crus II	4.57 (4.180)	4.62 (4.220)	4.53 (4.140)	-1.9244
	[4.092, 4.834]	[4.090, 4.906]	[4.024, 4.828]	[-0.1793, 0.1133]
Lobule VIIB	4.99 (4.563)	5.10 (4.662)	4.88 (4.461)	-4.4009
	[4.215, 4.969]	[4.189, 5.000]	[4.192, 4.989]	[-0.1286, 0.1231]
Lobule VIIIA	4.93 (4.511)	4.96 (4.532)	4.91 (4.490)	-0.9231
	[4.166, 4.901]	[4.122, 4.899]	[4.170, 4.942]	[-0.0915, 0.1329]
Lobule VIIIB	5.04 (4.605)	5.01 (4.578)	5.07 (4.632)	1.1756
	[4.094, 4.888]	[3.982, 4.884]	[4.138, 4.951]	[-0.0941, 0.1949]
Lobule IX	4.89 (4.467)	4.97 (4.540)	4.82 (4.402)	-3.0854
	[3.129, 4.941]	[3.042, 4.903]	[3.171, 5.024]	[-0.1511, 0.2969]
Lobule X	3.96 (3.624)	3.83 (3.501)	4.10 (3.752)	6.9174
	[3.129, 4.941]	[3.042, 4.903]	[3.171, 5.024]	[-0.1511, 0.2969]

Intracranial cavity segmentation[4]

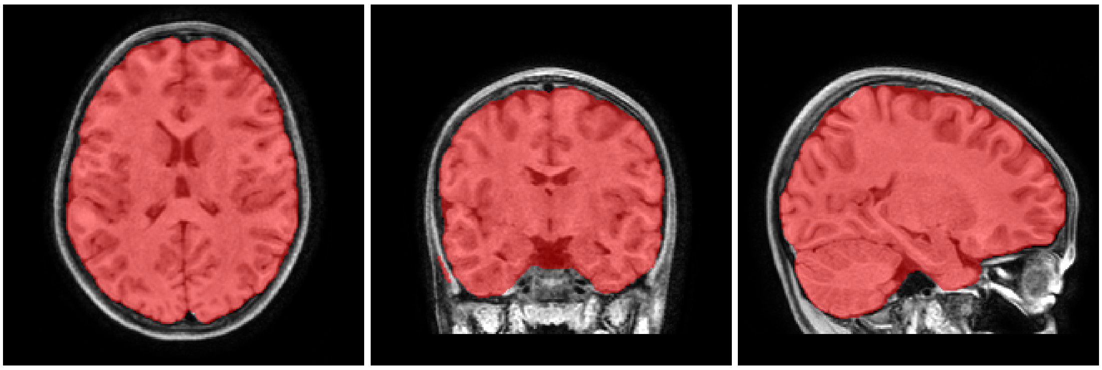

Lobule segmentation

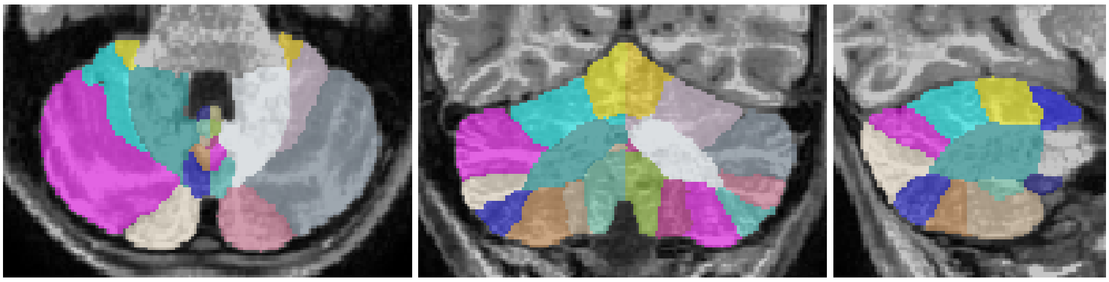

Tissue segmentation

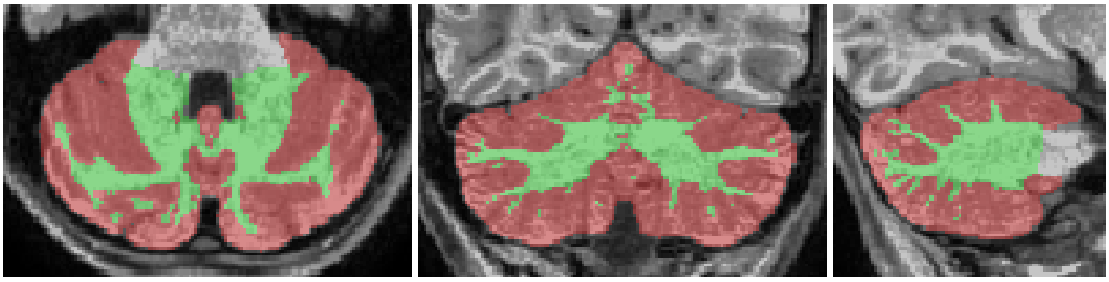

Cortical thickness

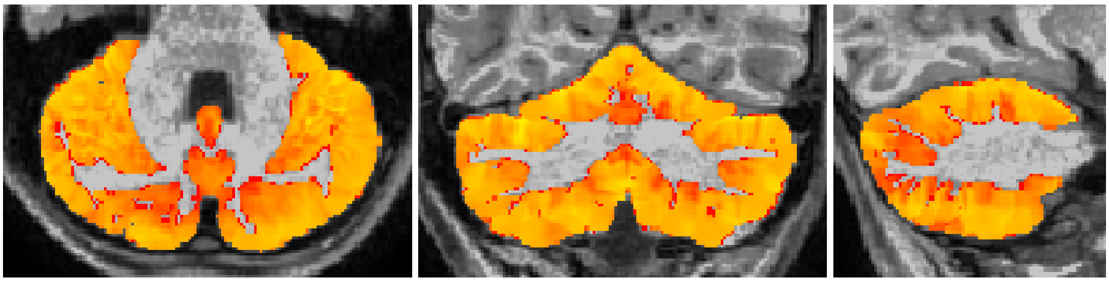

Supplement: Supplementary file 1 [file diagnostics-16-02085-s001.zip › Supplementary Material S2.pdf]
