# Supplementary material for: Multiplatform Morphometric Profiling of Whole-Brain, Cerebellar Subregional, and Thalamic Nuclei Alterations in Pediatric Migraine Without Aura
Source: Diagnostics (Basel). 2026 Jul 3;16(13):2085. doi: 10.3390/diagnostics16132085 (PMC13360080; doi:10.3390/diagnostics16132085)
Supplement: Supplementary file 1 [file diagnostics-16-02085-s001.zip › Supplementary Material S3.pdf]

Supplementary Material 3. Command-line implementation of the FreeSurfer-based thalamic nuclei segmentation and quality-control pipeline

The following command-line workflow summarizes the computational pipeline used for thalamic nuclei segmentation, including DICOM-to-NIfTI conversion, whole-brain reconstruction, Bayesian atlas-based thalamic nuclei segmentation, and export of segmentation outputs for anatomical verification and quality control.

Step 1. Launch FreeSurfer Docker container

```
docker run -it --rm \
  -v /path/to/license.txt:/usr/local/freesurfer/license.txt \
  -v /path/to/project_directory:/data \
  freesurfer/freesurfer:7.4.1 \
  bash
```

Step 2. Extract DICOM archive

```
cd /data
unzip subject_001.zip -d subject_001
exit
```

Step 3. Convert DICOM files to NIfTI format

```
sudo apt update
sudo apt install -y dcm2niix
mkdir -p /path/to/project_directory/nifti
dcm2niix -z y \
  -o /path/to/project_directory/nifti \
  /path/to/project_directory/subject_001
```

Step 4. Re-enter FreeSurfer container

```
docker run -it --rm \
  -v /path/to/license.txt:/usr/local/freesurfer/license.txt \
  -v /path/to/project_directory:/data \
  freesurfer/freesurfer:7.4.1 \
  bash
```

Step 5. Perform whole-brain reconstruction

```
export SUBJECTS_DIR=/data/subjects
mkdir -p $SUBJECTS_DIR
recon-all \
  -s subject_001 \
  -i /data/nifti/*.nii.gz \
  -all
```

Step 6. Install MATLAB Compiler Runtime (if required)

```
fs_install_mcr R2019b
```

Step 7. Perform thalamic nuclei segmentation

```
segmentThalamicNuclei.sh subject_001
```

Step 8. Export segmentation outputs for visualization

```
mkdir -p /data/slicer
mri_convert \
  /data/subjects/subject_001/mri/T1.mgz \
  /data/slicer/T1.nii.gz
mri_convert \
  /data/subjects/subject_001/mri/ThalamicNuclei.v13.T1.FSvoxelSpace.mgz \
  /data/slicer/ThalamusNuclei.nii.gz
```

Step 9. Verify exported files

```
ls /data/slicer
```

Expected output

```
T1.nii.gz
ThalamusNuclei.nii.gz
```

The exported NIfTI files were subsequently imported into 3D Slicer for anatomical verification and visual quality control prior to volumetric analysis.
