# Supplementary material for: Multiplatform Morphometric Profiling of Whole-Brain, Cerebellar Subregional, and Thalamic Nuclei Alterations in Pediatric Migraine Without Aura
Source: Diagnostics (Basel). 2026 Jul 3;16(13):2085. doi: 10.3390/diagnostics16132085 (PMC13360080; doi:10.3390/diagnostics16132085)
Supplement: Supplementary file 1 [file diagnostics-16-02085-s001.zip › Supplementary Material S4.pdf]

Supplementary Material 4. Confirmatory ANCOVA analyses of morphometric findings that remained statistically significant after primary FDR correction

Analysis family	Morphometric parameter	Side	Covariates included	ANCOVA F	Adjusted p-value	Adjusted q-value (FDR)
**Whole-brain morphometry**	Amygdala volume	Right	Age, sex, BMI, TIV	7.94	0.006	0.031
**Whole-brain morphometry**	Amygdala volume	Left	Age, sex, BMI, TIV	7.21	0.008	0.034
**Whole-brain morphometry**	Anterior insula cortical thickness	Right	Age, sex, BMI	6.86	0.010	0.037
**Whole-brain morphometry**	Anterior cingulate gyrus cortical thickness	Left	Age, sex, BMI	6.38	0.013	0.041
**Whole-brain morphometry**	Precuneus cortical thickness	—	Age, sex, BMI	5.72	0.018	0.046
**Whole-brain morphometry**	Thalamus volume	Right	Age, sex, BMI, TIV	4.98	0.027	0.049
**Cerebellar subregional volume**	Lobule VI volume	Right	Age, sex, BMI, TIV	5.96	0.016	0.043
**Cerebellar subregional volume**	Lobule VI volume	Left	Age, sex, BMI, TIV	5.54	0.020	0.047
**Cerebellar gray matter volume**	Lobule VI gray matter volume	Right	Age, sex, BMI, TIV	8.47	0.004	0.020
**Cerebellar gray matter volume**	Lobule VI gray matter volume	Left	Age, sex, BMI, TIV	7.68	0.007	0.025
**Cerebellar cortical thickness**	Lobule VIIA (Crus I) cortical thickness	Right	Age, sex, BMI	7.36	0.008	0.033
**Cerebellar cortical thickness**	Lobule VIIA (Crus II) cortical thickness	Right	Age, sex, BMI	6.49	0.012	0.045
**Thalamic nuclei volume**	Ventral anterior nucleus (VAN)	Right	Age, sex, BMI, TIV	5.83	0.017	0.043
**Thalamic nuclei volume**	Ventral posterolateral nucleus (VPLN)	Right	Age, sex, BMI, TIV	6.63	0.011	0.038
**Thalamic nuclei volume**	Pulvinar nucleus (PN)	Right	Age, sex, BMI, TIV	7.72	0.006	0.028
**Thalamic nuclei volume**	Pulvinar nucleus (PN)	Left	Age, sex, BMI, TIV	6.96	0.009	0.035
**Thalamic nuclei volume**	Mediodorsal nucleus (MN)	Right	Age, sex, BMI, TIV	8.31	0.005	0.022
**Thalamic nuclei volume**	Mediodorsal nucleus (MN)	Left	Age, sex, BMI, TIV	6.18	0.014	0.040

Values represent ANCOVA-adjusted group comparisons. Only morphometric parameters that remained statistically significant after the primary analyses and subsequent Benjamini–Hochberg false discovery rate (FDR) correction were subjected to confirmatory ANCOVA reanalysis. Age, sex, and body mass index (BMI) were included as covariates in all models, whereas total intracranial volume (TIV) was additionally included as a covariate for volumetric measurements. ANCOVA-derived p-values were subsequently corrected using the Benjamini–Hochberg false discovery rate procedure within each corresponding analysis family. All morphometric parameters listed in this table remained statistically significant following covariate adjustment and subsequent FDR correction, confirming the robustness of the primary morphometric findings.
